# Barriers and facilitators of accessing primary healthcare for patients with severe mental illness: a mixed-methods systematic review using framework synthesis

**DOI:** 10.1186/s12888-025-07565-x

**Published:** 2025-11-27

**Authors:** Elena Malogianni, Modhie F. Alenezi, Laoise Renwick

**Affiliations:** 1 Division of Nursing, Midwifery & Social Work, University of Manchester, Manchester, UK; 2 Avon and Wiltshire Mental Health NHS Trust, Bath, UK

**Keywords:** Serious mental illness, Severe mental illness, Barriers, Facilitators, Views, Attitudes, Primary health care, Primary care, General practice

## Abstract

**Background:**

Serious Mental Illness (SMI) is associated with significant physical health inequalities and a shortened life expectancy of approximately 20 years compared to the general population. Access to primary care (PC) services is crucial for addressing the complex healthcare needs of individuals with SMI.

**Aims:**

To identify and analyse the barriers and facilitators that individuals with SMI encounter when accessing PC.

**Methods:**

Five electronic databases (PsycInfo, EMBASE, Medline, CINAHL, Web of Science) were searched to identify primary studies, in the English language, that investigated factors affecting SMI patients’ access to PC, published from inception to November 2022 and again in May 2024. All studies were assessed for their quality using the JBI critical appraisal tools (i.e. JBI checklists for analytical cross-sectional studies and qualitative research) and extracted data were synthesised using framework synthesis and a convergent integrated mixed-methods approach.

**Results:**

38 studies were found eligible and were included in the synthesis. The identified barriers included, among others, the SMI-related stigma leading to the lack of holistic care, the absence of continuity and rapport in the patient-clinician relationship, the short length of appointments, the fragmented communication between PC and mental health (MH) services, clinicians’ inadequate training on SMIs & patients’ socioeconomic circumstances. Conversely, reported facilitators were the education and active involvement of patients in healthcare-related decisions, the presence of long-term trusting patient-clinician relationships, the implementation of structured annual reviews, as well as the presence of professional contacts or social networks that can support patients with the coordination of their healthcare, accompany them to appointments and provide advocacy.

**Conclusions:**

The review highlights the urgent need for strategies that will reduce the persistent stigma around SMIs, enable care continuity, promote a shared-care approach between PC and MH services and offer adequate support to patients to access healthcare when needed. Future research should focus on evaluating the effectiveness of such approaches and expanding the evidence base in this critical area.

**PROSPERO registration:**

The review was registered in PROSPERO with registration number CRD42023430415.

**Supplementary information:**

The online version contains supplementary material available at 10.1186/s12888-025-07565-x.

## Background

People with serious mental illness (SMI) have an increased risk of developing physical illnesses, reduced access to healthcare and premature mortality reducing their lifespan by approximately 20 years [1–6]. Health inequalities and premature mortality have been reported in research for over 50 years [7, 8] and despite the growing body of research on this subject and the progress in healthcare over recent decades, the poor physical health of people with SMI has not shown signs of improvement [9, 10]. The mortality gap for people with SMI not only continues to exist but, in fact, the research available indicates it is increasing in size [11, 12]. Estimates show premature mortality is up to 3.5 times greater than in the general population [3, 4, 13, 14]. These excess rates exceed mortality gaps for both diabetes [15] and smoking [16]. The fact that individuals with SMI die much younger than those without a serious psychiatric condition, but with similar physical illnesses, reflects profound social discrimination and one of England’s most substantial health disparities [17, 18]. While improving access to healthcare is a critical component in addressing disparities in physical health outcomes for individuals with SMI, broader social determinants—including socioeconomic status, housing, employment, and social inclusion—also significantly influence overall health and well-being. As highlighted by Marmot [19], structural inequalities contribute to these disparities, underscoring the need for systemic interventions beyond healthcare access alone.

Among those with SMI, the majority of human loss is associated with poor physical health commonly including cardiovascular conditions [13, 14], respiratory illnesses, stroke and cancer [20]. However, the high rates of physical comorbidity, are linked with limited access to care and poor clinical management, further contributing to healthcare disparities. The development of the Lester Tool, which aims to improve the cardiovascular health assessments of SMI patients [21], systematic reviews (SRs) exploring the impact of physical exercise [22] and randomised controlled trials (RCTs) examining the effects of modifiable risk factors, such as smoking [23, 24] and metabolic syndrome [25], are part of the expanding literature demonstrating positive health outcomes from both discrete and health promotive interventions. However, the majority of this recent activity concentrates on initiatives that aim to enhance the promotion of health by encouraging changes in individual behaviour, such as increasing physical activity. Although these are undoubtedly valuable, they may have limited reach and impact on broader healthcare access [26].

A substantial knowledge gap exists when it comes to systemic strategies that could improve access to and quality of care provided and the physical health outcomes of these individuals on a broader scale. The management of patients with SMI presents a multifaceted challenge, with primary care (PC) typically being considered responsible for addressing the majority of their physical health issues [27] and secondary mental health (MH) services accountable for managing their MH concerns [28]. Although, on many occasions, this allocation becomes much less clear, PC plays an important role as the cornerstone of healthcare for this population [28], with up to 30% exclusively seeking care in the GP setting in the UK [29]. Unlike secondary MH services, where there are often significant delays before seeing a specialist, where GP services are readily available and more easily accessible, they can offer more timely assistance, potentially contributing to better health outcomes [30]. Furthermore, they facilitate preventative healthcare with the promotion of positive lifestyle choices, aiming to minimise risk factors for physical health conditions [31]. PCs role in managing chronic physical illnesses should also not be understated, as the expertise of primary care professionals (PCPs) is critical in ensuring that SMI patients receive comprehensive care that goes beyond having a mental illness.

Despite this, disparities continue in the provision and receipt of healthcare services across primary care services. People with SMI are more likely to be treated in the emergency department for conditions that could be more easily managed in PC settings [32]. Individuals with SMI also have greater medical complexity, require longer consultations and have a higher need for follow-up care when compared with general populations attending PC [33, 34] while few receive this degree of care [35–37]. Given its substantial role in managing the physical health needs of SMI patients, it becomes apparent that PC is strategically positioned to contribute significantly to bridging this mortality gap [37–39]. Certain structural aspects of healthcare, such as better integration between primary and secondary services and enhanced staff training, can be more easily identified and targeted [34, 40, 41]. Soft elements, however, such as culture, attitudes and perspectives are less easily discerned but may be equally salient for their potential impact on embedding interventions into healthcare systems to improve the management of physical health conditions for people with SMI.

Despite the extensive research investigating access to primary healthcare for patients with SMI, no review systematically examining the topic has been conducted to date. The aim of this systematic review is, therefore, to provide a robust and in-depth examination of the barriers and facilitators to accessing care in PC settings exploring the views and perspectives of both recipients and providers of care and treatment. We employed a theoretical framework of healthcare access which allows a multi-level perspective of access and permits identification of relevant determinants that can impact access from these perspectives.

## Methods

The review was registered in PROSPERO (CRD42023430415) and was conducted following the guidelines of Preferred Reporting Items for Systematic Reviews and Meta-Analyses (PRISMA) 2020 statement [42] (Tables s1 & s2).

### Eligibility criteria

We sought to identify studies reporting primary evidence of the views and experiences of people with SMI and PCPs regarding patients’ access to PC services. Studies with qualitative, quantitative or mixed-method designs were included in order to enhance the review’s findings [43]. We included quantitative, qualitative and mixed methods designs allowing exploration of the level of agreement between the different types of findings and the determination of the degree to which qualitative and quantitative data could address the various aspects from the perspective of key stakeholders [44]. We included peer-reviewed journals with no restriction on the date the study was completed. We excluded unpublished data, non-English papers, conference proceedings, dissertations and secondary resources to ensure that all included studies met peer-reviewed publication standards, thereby enhancing methodological rigour and the reliability of findings. Studies with a patient population of under 14 were also excluded. Inclusion/exclusion criteria are described in Table s5 in the Supplementary material.

### Search strategy

Five electronic databases (Embase, Medline, Web of Science, PsycInfo and CINAHL) were systematically searched during initial searches from inception till the 26^th^ of November 2022. Eighteen different strategies were developed and tested on all five databases. Consensus on the final search strategy was achieved among the study team following guidance from a specialist systematic review librarian at the University of Manchester. The search strategy was defined in four components utilising the PICo tool (Population, phenomenon of Interest & Context) [45]. These comprise: (1) SMI patients, (2) access, (3) PC and (4) views, observations or experiences

According to NHS England, SMI refers to chronic and debilitating mental health conditions and is described variably across the literature [46]. For the purposes of this review, SMI was defined in line with standard clinical classifications as encompassing schizophrenia, bipolar disorder, and other psychotic disorders [47]. Studies focusing exclusively on common mental disorders, such as depression or anxiety, without co-occurring SMI were excluded. Primary care was defined as first-contact, accessible, and continuous healthcare services, typically delivered by general practitioners, nurses, and other allied health professionals within community or outpatient settings [48].

In each category, similar terms were combined with the Boolean operator “OR” and the four components with “AND” (see Table s3 for the complete search strategy). No chronological limits or restrictions based on the country of origin were set for the searches. The process was completed by reviewing the references of the included studies for potentially relevant articles. Deduplication was managed in the Endnote reference manager [49] before being imported to Covidence [50]. Two reviewers (EM, LR) independently carried out the initial screening of studies by title and/or abstract. Reviewer decisions were compared within Covidence, which automatically flagged conflicts for resolution. Any disagreements were discussed in detail until consensus was reached. In cases where consensus could not be reached, a third reviewer was consulted to make the final decision. Full-texts were obtained, and the full-text screening took place by two independent reviewers (EM, MA) and the involvement of a third researcher (LR) where agreement could not be reached on conflicts. The searches were repeated on the 1^st^ of May 2024 in order to include any recently published, relevant papers prior to publication and the screening process was repeated.

### Data extraction and quality appraisal

During the planning phase, we considered the possibility that our data might not fully align with Levesque’s framework for healthcare access [51], so we conducted inductive coding first to identify themes that did not neatly fit within its existing components. This was followed by deductive coding to ensure a structured application of the framework while allowing flexibility for additional insights. The framework was trialled using eight selected papers and was found to comprehensively capture all necessary components without requiring modifications. This approach enabled a more nuanced analysis, ensuring that the framework supported rather than restricted the synthesis of evidence.

Data were extracted to an Excel sheet developed purposely and piloted prior to the review. Data extraction was commenced on the 30^th^ of May 2023 and qualitative and quantitative outcome data were extracted simultaneously including year of publication, country, setting, study design, primary aim, participant characteristics, inclusion criteria, sampling, data collection and analysis methods.

We elected to use the Joanna Briggs Institute (JBI) tools to evaluate the quality of included studies based on guidance from JBI, given that methods to effectively conduct critical appraisal in mixed-methods SRs (MMSRs) seem to lack unified agreement [52, 53]. The appraisal of quantitative studies was conducted using the JBI critical appraisal tool for analytical cross-sectional studies [54], while the JBI checklist for qualitative research was implemented to appraise the remainder of the studies [55]. The checklists have been widely piloted and are both valid and coherent in comparison with similar tools [56]. Studies were not excluded based on quality assessment as empirical evidence is lacking to support exclusion based on quality criteria [57].

The data extraction and quality appraisal were conducted independently by two researchers, with results subsequently compared for consistency. Any discrepancies were discussed collaboratively until agreement was achieved; consensus was reached through iterative discussion and cross-checking of extracted information against the primary sources to ensure accuracy and transparency in the final dataset.

### Data analysis and synthesis

A narrative synthesis of qualitative and quantitative studies was conducted using deductive and inductive thematic methods within Levesque’s existing conceptual framework of Access to Healthcare [51, 58], which was identified as the most up-to-date and relevant to the review [59]. To evaluate the robustness of our synthesis, we examined the findings within the analysis contained in each component of the framework when a) studies with weak quality scores were removed and b) studies with moderate–weak scores were removed. We then assessed the contribution of each individual piece of evidence to the consistency of descriptions within our synthesis to examine whether different pieces of information were compatible with the overall synthesis.

**Fig. 1:**
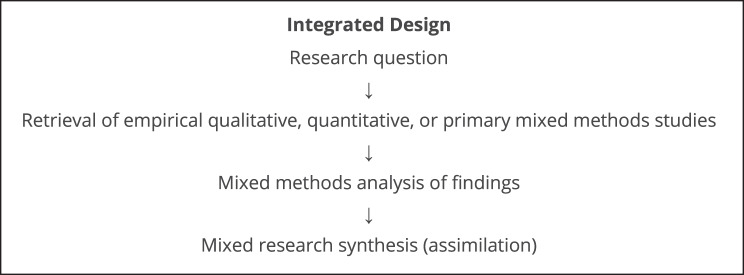
The integrated design for MMSR [60]

We utilised a convergent integrated approach (Fig. 1), where all studies were analysed simultaneously using the same methods and results discussed collectively [53]. This approach, which has been recommended by the updated JBI guidance for the conduct of MMSRs [61], assumes that the methodological differences between qualitative and quantitative studies are diminished because both are perceived as generating findings that can be easily transformed to one another as they address the same research objectives. It involves transforming data, by either turning qualitative data into quantitative (quantising) and vice versa (qualitising), enabling their synthesis [44]. In concordance with JBI guidance, quantitative data were converted to “textual descriptions”, such as narratives and themes, to enable their synthesis with qualitative findings. Mixed-method primary studies were disaggregated into quantitative and qualitative data, to allow their synthesis with the rest of the studies [44], and each component was analysed based on its relevance to the research question.

Data integration was achieved through a combination of deductive coding, aligning findings with the conceptual framework of access to healthcare, and inductive coding, capturing emergent themes across studies. This allowed for the identification of convergent and divergent evidence across study types, highlighting complementary insights and enhancing the robustness of the synthesis. This integrated approach ensured that both qualitative and quantitative evidence contributed meaningfully to the synthesis, enhancing methodological coherence and the explanatory power of the mixed-methods review.

## Results

### Study characteristics

**Fig. 2:**
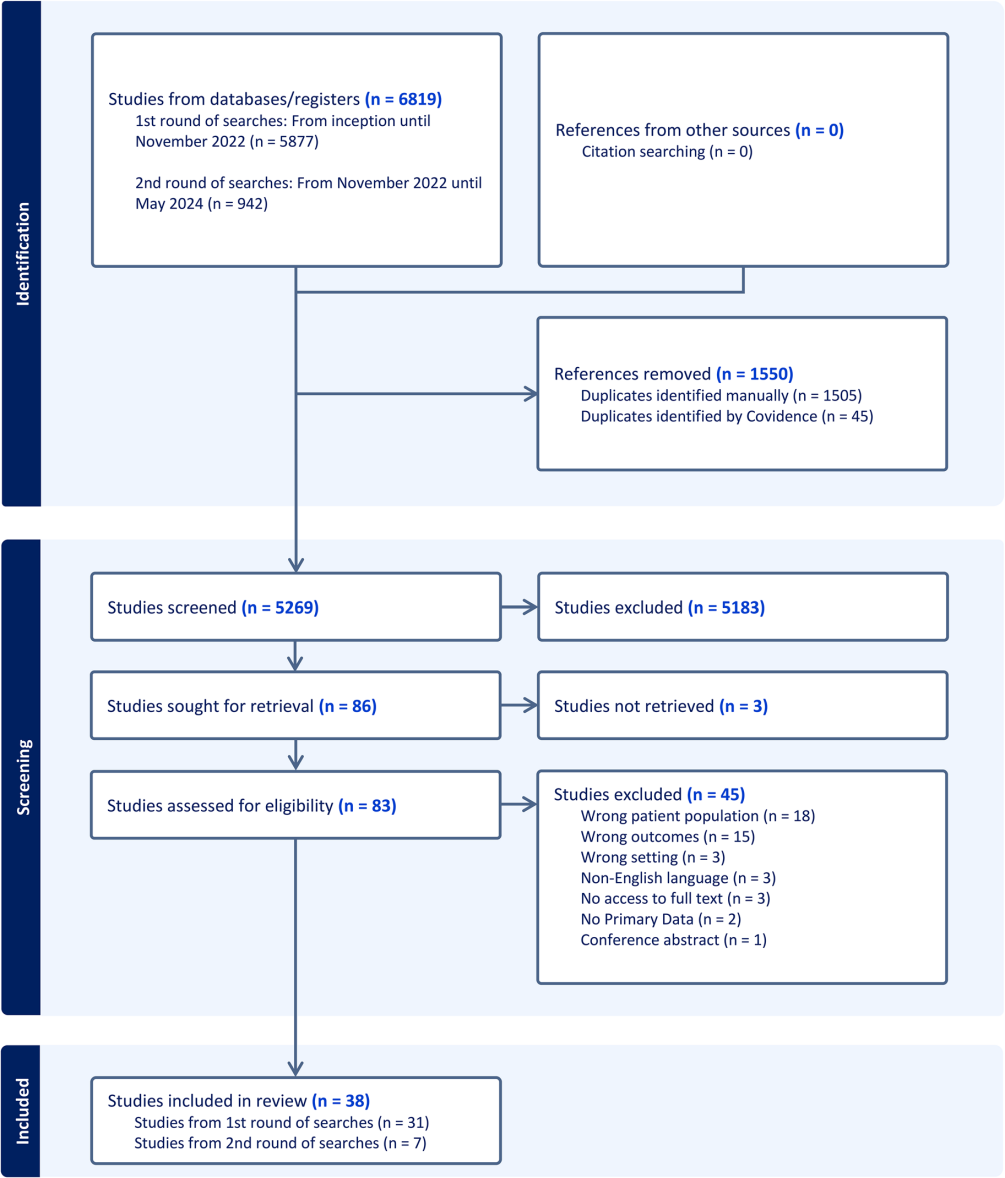
Prisma flow diagram of the review

The PRISMA study flow diagram depicts how papers were selected for inclusion in this review (Fig. 2). 31 articles met the inclusion criteria from initial searches with 7 from subsequent searches (Additional File 1). Studies included the views of people with SMI exclusively (*n* = 4), PC professionals (*n* = 8) or mixed samples (*n* = 12). The remainder (*n* = 14) included MH professionals, other healthcare professionals, family members or carers alongside our population of interest and, in these instances, only the relevant data were extracted.

The earliest study was conducted in 1997 though the majority were conducted in the last 8 years. The majority were conducted in high-income countries, mainly UK (*n* = 15), USA (*n* = 5), Australia (*n* = 4) and Denmark (*n* = 3), although the results represented 14 different countries from all 5 continents. Studies used qualitative methodologies employing semi-structured interviews (*n* = 17), focus groups (*n* = 2) or a combination of interviews, discussion groups and/or field notes (*n* = 6). Quantitative surveys (*n* = 10) and mixed methods studies (*n* = 3) made up the remainder.

In total, views from 2765 patients with SMI from 26 studies and 3587 PCPs from 27 studies were included in the review; out of those, results from 456 patients and 1834 PCPs originated from quantitative studies. Approximately 77.7% of people with SMI had a diagnosis of either schizophrenia, bipolar disorder, or schizoaffective disorder, based on the studies that reported diagnosis. 40.5% (*n* = 1108) identified as female, and the age range of the total patient population was between 18 and 77 years old. Healthcare professionals comprised mainly of GPs (*n* = 2636, 73.5%) and practice nurses (*n* = 918, 25.6%) and from papers reporting sex, 62.1% were female (*n* = 2227). There were few studies reporting the age range for professionals, which was between 20 and 78 years old.

### Quality appraisal

The majority of qualitative studies fulfilled most of the criteria on the JBI appraisal tool and were assessed to be of high to moderate quality; they implemented consistency in reporting the research methodology, outcomes, data collection, analysis and interpretation of results, while discussions and conclusions had clear links to analysis and interpretation of findings. However, almost all qualitative studies were assessed to have weaker elements regarding their theoretical standpoint and reflexivity. Variation in quality was evident among quantitative studies, which were examined for their contribution to the synthesis to determine the weight of evidence from these sources. With the exception of one paper [62], no other quantitative study was found to fulfil all eight standards as described by the JBI tool. Risk of measurement bias was identified in many of them as they appeared to be lacking clear reporting on whether valid and reliable methods were used for the measurement of the exposure/outcome studied. Quality appraisals can be found in Tables s6-7.

### Synthesis

We synthesised patient and professional views together and these are reported using Levesque’s [51] framework of patient-centred access to healthcare to allow a comprehensive understanding of factors that influence access or identify aspects that could be leveraged to enhance access. Emergent themes are discussed in detail below (Additional file 2, Fig. 3) and accompanying quotes that support findings are also provided (Boxes s1-10 in Supplementary material).

**Fig. 3:**
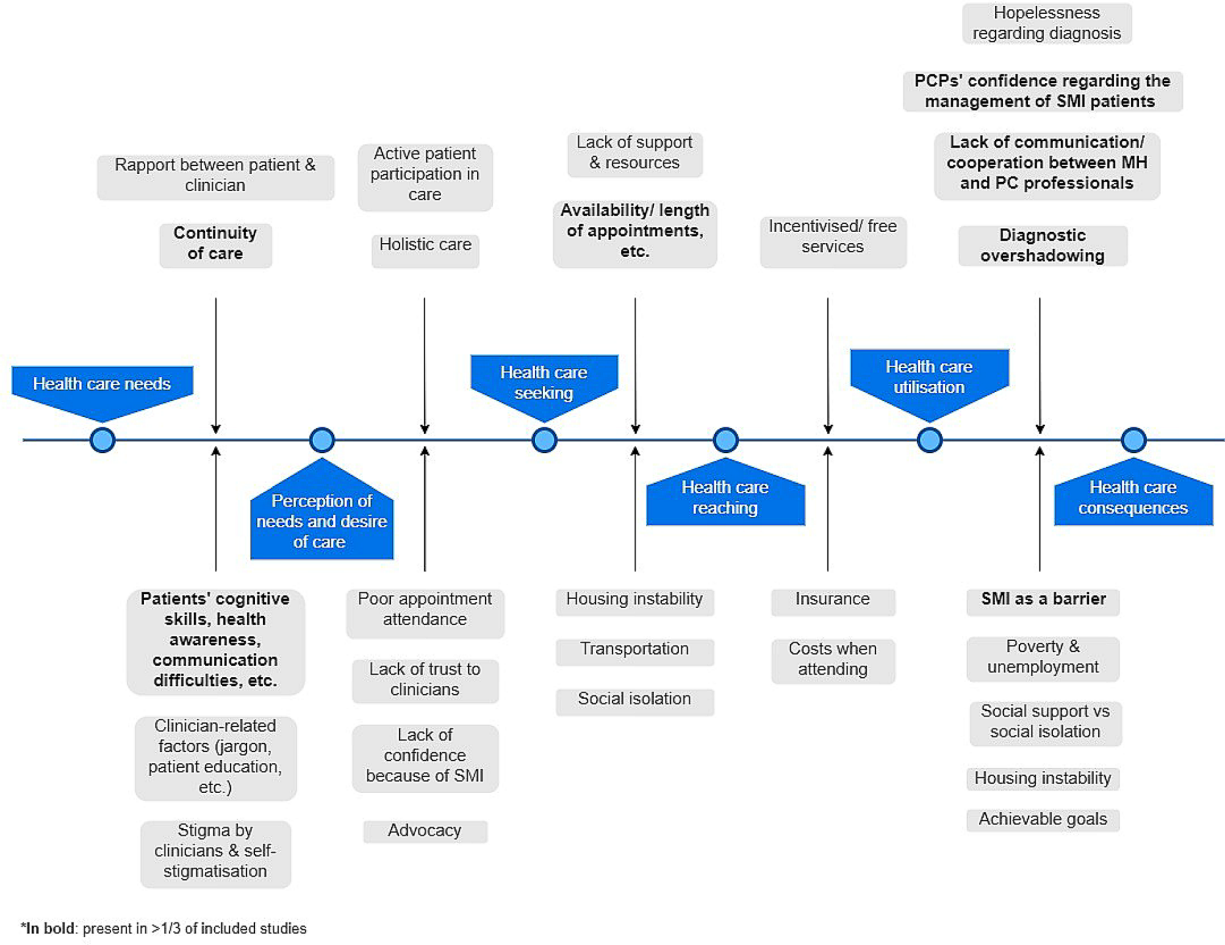
Emergent themes

#### Approachability

Continuity of care, and how patients perceived consultations consistent with their needs, was reported frequently as a crucial aspect of receptive and responsive services [28, 62–77]. Interpersonal and longitudinal continuity was considered essential for high-quality care in reports from both service users and professionals allowing patients to develop confidence and trust in clinicians [63, 68, 78, 79] and counteract other barriers to access [72]. This interpersonal continuity, from professionals’ perspectives, enabled providers to approach patients in an individualised way based on their needs [69, 78], while patients reported that knowing their PCP allowed them to engage more actively in their own care, thus, promoting shared decision-making and improving experiences of care [28, 68].

Rapport and understanding between professionals and service users emerged as an important element of clinical contacts that influenced perspectives of access from multiple stakeholder groups. Professional behaviours detrimental to developing rapport included poor communication skills [63, 80] and lack of respect and empathy [62, 79, 81]. Encounters in which professionals were detached and bureaucratic made relationships feel “business-like (sic) and impersonal” [66, 80]. There was also strong evidence that stigmatising attitudes significantly impacted the approachability of services from the perspective of patients. Some PCPs reported being wary of people with SMI [65, 71, 82], which affected their ability to play an active role in patients’ healthcare [68, 83]. PCPs’ prejudice and stereotypical perceptions against SMI, cultivated a sense of inferiority [84] and, based on service users’ views, reduced their motivation [85] and confidence in accessing PC [72].

#### Acceptability

Acceptable care was provided on the basis of holistic and patient-centred principles [62, 68] increasing trust and promoting a positive view of self-identity among service users. Patients strongly held the opinion that they should be considered as a whole and be investigated and treated for both their physical and MH issues [63, 80, 85]. Service users expressly stated their desire for active participation in their own care and to be treated as equals, yet often experienced the opposite. GPs addressing conversations during consultations only with the people accompanying them [71, 81] and ignoring their requests regarding treatment and medication changes [28, 79, 80, 86] were specific examples. Conversely, when professionals allowed service users to participate in their care and offered psychotherapy and alternative therapies, patients’ hope for recovery was improved [28].

#### Availability and accommodation

The most common factors related to availability were associated with time constraints. Both patients and providers found the consultations in PC too short to allow service users to adequately explain their symptoms [75, 87, 88] and, thus, negatively impacted the provision of holistic care [62, 65, 81]. Moreover, due to the lack of time, PCPs reported finding it difficult to educate and encourage the patients and promote the engagement required [62, 68, 74, 88–90]. This barrier was confirmed by 22.1% of GPs in the study by Waterreus and Morgan [74], while, according to Pitman et al. [91], almost a quarter of patients attributed their inability to receive the help they need in order to make lifestyle changes, to time pressures [91].

The large demands on the healthcare system and the overworking of PC staff were also considered important barriers by PCPs [63, 77, 78, 82]. The overall system pressure, combined with the cuts in funding and the lack of adequate human and practical resources forced professionals to act within limits and compromised the provision of high-quality healthcare [62, 64, 89]. Patients admitted that the long waits for appointments were a common problem they faced [65, 72, 76], which often resulted in them having to negotiate access if they were not acutely unwell [28]. The crowded waiting areas and long waiting before appointments added to their anxiety [28, 65, 66, 81, 82], the overall hurried atmosphere [66, 81], the excessive administrative processes, the necessity to disclose confidential information to non-clinical staff [65] and the lack of awareness of the available services and resources [66] were also discussed as barriers by patients.

When provided, service users appreciated the increased length of intervention appointments, which made care feel more holistic [68, 81, 86], findings supported by Waterreus and Morgan [74]. Furthermore, the implementation of structured routine reviews, such as annual reviews, was supported by both service users and PCPs [28, 63, 64, 89], with 96% of nurses [92] and 89% of patients [91] endorsing their use. The ease of access for SMI patients should be reinforced as much as possible [71] by accommodating their needs regarding location and timing [68, 71, 81, 84], adapting interventions based on their understanding [63] and enabling them to have easy access to all relevant information regarding PC access [66].

#### Affordability

Only three studies reported on factors associated with the affordability of PC services and interventions. Burton, Osborn [65] and Bosanquet, Gilbody [62] described in their findings the negative impact of the elimination of incentivised or free services, which directly restricts patients’ access to different healthcare services. In the study by Spooner [81], the participants only accessed GPs who bulk billed and the ones with access to preventive programs explained, that they could participate only because these were free or heavily subsidised.

#### Appropriateness

One of the most commonly reported concepts was that of interprofessional communication, with both providers and service users discussing the importance of timely and collaborative communication between PCPs and MH professionals. PCPs considered the overall lack of collaboration and information-sharing between the two as a significant barrier [62, 73, 75–77, 84, 89, 92, 93] and discussed their inconvenience in establishing a point of contact in MH services [62]. This lack of communication was also reported by service users, who often felt helpless and confused [64, 70, 76, 84, 94].

Different approaches, addressing the collaboration of PCP and MH professionals, have been described by both providers and service users as facilitators of access for patients. Bindman, Johnson [95] found that GPs were more willing to take part in shared care of SMI patients if there was better and more timely communication between specialities, while Waterreus and Morgan [74] discovered that 73.1% of GPs were willing to take responsibility of patients’ psychiatric care if MH support was readily available. The themes of effective communication, continuity, and exchange of knowledge between PC and MH services were particularly strong and expressed by both patients and PCPs in multiple other studies [62, 64, 65, 76, 77, 84, 85, 90, 93, 96].

Clinicians’ lack of adequate experience treating people with SMI was a theme commonly discussed by PCPs, often resulting in a lack of confidence on their side [28, 64, 68, 74, 82, 85, 92, 96]. The shortage of training on MH conditions and the need for appropriate education, that would support the professional development of PC staff, were reported by both patients and PCPs [62, 63, 65, 82]. Moreover, they both agreed that one professional should be overall responsible for patients’ care and the coordination of interprofessional communication, as this would allow for flexibility, clarity of roles and improved liaison between primary and secondary care [64, 77, 96].

Another particularly common theme was that of diagnostic overshadowing, with patients reporting that their physical problems were often ignored or attributed to their MH illness and not taken seriously by clinicians [63–66, 72, 75, 76, 80–82, 85], while both groups felt the main focus of care was on the patients’ MH issues [89]. Service users often disclosed that they were not treated equally and had to delay treatment of a physical health problem prior to a MH crisis despite recognising the need for care [66, 80, 82, 87], while some clinicians significantly reduced the number of referrals for SMI patients, due to their belief that they would not easily be able to participate in the available services [85]. The MH-related stigma was also reflected in clinicians’ perception of SMI as a “life sentence”, cultivating hopelessness for patients who believed their diagnosis was unchangeable and ending up perpetuating their self-stigma [71, 82]. In contrast to this view, the acknowledgement of the potential to improve and the defition of achievable goals were considered by patients as important factors in increasing their satisfaction and improving their access to services [69].

#### Ability to perceive

Both patient- and professional-related factors were found to have an impact on accessibility. PCPs reported that SMI patients’ cognitive and communication abilities were often reduced, making it difficult for clinicians to properly assess them [28, 64], while both groups discussed the patients’ lack of understanding of their physical illnesses and their relevant management and side effects as additional barriers [65, 68–70, 75, 76, 79, 81, 89, 90, 94]. An additional impediment to access was related to SMI patients’ normalisation of poor physical health and lack of self-management skills [72], while their difficulty in completing all documentation required for their access also negatively affected their consultations [68]. On the other hand, patients considered the lack of consideration of their abilities by clinicians and the use of overcomplicated explanations to have a negative impact on their ability to perceive [72].

Patients’ education and the provision of support in order to improve their health literacy were both reported to positively influence their understanding and increase their engagement with PC [66, 67, 81, 87, 94]. Moreover, service users admitted that they appreciated being accompanied to appointments as they often lost concentration, could not fully understand all information provided and struggled to describe their symptoms accurately [87]. Finally, PCPs expressed the need to adapt interventions according to patients’ understanding and interests and be able to provide them with alternatives, in order to best address their needs [63, 76].

#### Ability to seek

Providers argued that one of the impediments in patients’ access to PC is associated with their poor attendance of appointments and supported that SMI patients are less likely to seek care regarding their physical health [75, 89]. Patients, on the other hand, explained that previous negative experiences with clinicians and their lack of trust towards them, especially when their MH is poor, could have a negative impact on their care-seeking behaviour, including their tendency to delay treatment [66, 67, 81]. They also argued that their reluctance to look for help is a result of their MH illness itself, as they tend to lack the confidence needed in order, for example, to book an appointment when they are unwell [28, 77]. Only one study highlighted factors that facilitate help-seeking [72]; these included the presence of social networks, which enabled patients to navigate and negotiate access to healthcare, and the provision of support and advocacy, such as being accompanied to appointments or having their family asking for help when their SMI has deteriorated.

#### Ability to reach

Multiple different factors were found to affect patients’ ability to reach PC, most of which were related to patients’ social circumstances. Housing instability was reported by service users as one of them, because of its negative impact on the development and sustainability of long-term relationships with local PCPs [66]. Social isolation and limited transport options were also problems faced by patients with SMI when trying to reach care [79–81], while according to Pitman, Osborn [91], 35% of service users reported their difficulty travelling as a barrier to obtaining help from PC. Many of the above issues could be resolved with the availability of support of patients’ transportation to their appointments [66, 81] and the use of home visits and home services, such as pharmacy deliveries of their medication [72].

#### Ability to pay

The studies that reported on barriers and facilitators regarding patients’ ability to pay for access to healthcare were limited, with very few associated factors identified. In the article by Mangurian, Giwa [88], PCPs mentioned patients’ lack of insurance as an impediment to access, while 79% (*n* = 19) of nurses in the paper by O’Brien and Abraham [92] considered the costs that could be incurred when attending appointments as additional barriers. According to DeCoux [66], the provision of financial support, that would cover the cost of services and medical supplies, would have a positive impact on patients’ access to PC.

#### Ability to engage

Service users’ ability to engage with their healthcare was associated with multiple different factors, both illness-related and based on social circumstances. PCPs supported that patients’ disengagement was, on many occasions, caused by the direct impact of their SMI, which made them less likely to attend appointments and comply with treatment compared to the average patient [28, 62, 65, 68, 74–76, 81, 86, 89, 90, 96]. This difficulty in adhering to appointments and treatment was, according to both patients and providers, related to their lack of motivation and low mood that presented as negative symptoms of their MH disorder [28, 65, 68, 75, 79, 84, 85, 88–90]. Moreover, PCPs supported that service users’ limited coping mechanisms and decreased ability to make changes independently had a significant negative impact on their ability to engage with PC [63, 90].

The lack of family and social support was reported by service users as an additional barrier, with them expressing fear of attending and organising appointments alone and having difficulty in explaining their concerns without advocacy [66, 80]. Their social isolation, poverty, unemployment, and limited access to transport additionally affected their ability to engage with providers [72, 75, 79]. Conversely, the presence of adequate support had a significant positive influence on patients, who appeared to be more willing to improve their physical health when they were appropriately supported, they could better understand their illness, attend appointments and adhere to treatment [28, 65, 72, 77, 81, 82, 89]. An additional facilitator proposed by service users was related to the provision of relevant and appropriate healthcare information by PCPs, which could improve their health literacy and allow them to feel empowered regarding their health [80]. Lastly, both patients and PCPs agreed that the definition of small and achievable goals in partnership with one another could positively affect their engagement and overall access to care [65, 76].

## Discussion

The findings of the review create a multifaceted picture of the factors that improve and compromise the quality of primary healthcare, as far as the physical health of people with SMI is concerned. Patients’ and providers’ experiences allowed examination of the topic from a dual perspective. Interpersonal therapeutic relationships, professional values that enhance therapeutic engagement and care organisation systems that negatively impact accessible primary healthcare services were prominent concerns for both stakeholder groups. Patients prioritised therapeutic relationships based on mutual understanding and trust; relationships were perceived to be heavily influenced by the interpersonal skills of providers and their ability to show respect and empathy. Conversely, a perceived lack of understanding and patience from PCPs negatively impacted patient-clinician interaction. This is not surprising, as multiple papers have recently discussed the crucial role that empathy and compassion can play in patient-clinician communication in all different clinical settings [97–100].

Patients and professionals converged in their views about the importance of continuity in care, with patients noting they often struggled to access PC due to the lack of long-term patient-provider relationships. When care continuity was ensured, service users were able to develop confidence in their clinicians, act on recommended interventions and overcome other possible barriers to access. Although this finding is confirmed by additional studies [101, 102], the current workload and the lack of GPs and funding in primary healthcare have hindered PCPs’ ability to build long-term connections with patients [103]. Patients desired equal partnership with PCPs, sharing decisions, particularly regarding medication decisions but reported limited influence over their healthcare in PC settings, which highlights the need for a more collaborative approach to treatment decisions. This problem is perpetuated by patients’ lack of understanding of illnesses and administrative processes, as well as the reduced cognitive & communication skills experienced by a significant percentage [104] making it difficult to navigate access when healthcare assistance is needed. Our findings align with Voorhees et al.‘s [105, 106] concept of ‘human fit’ in primary care access, which emphasises the interplay between patients’ abilities to seek care and healthcare providers’ capacity to make care accessible. This perspective reinforces the importance of fostering strong therapeutic relationships and ensuring continuity of care to improve access for individuals with SMI.

Importantly, holistic and patient-centred care is mentioned in multiple studies by both clinicians and service users, who emphasise the value of healthcare that addresses both physical and mental aspects and enables trust within the patient-provider relationship. We sought to gather views about access to PCP for patients with SMI assuming a focus on meeting physical health needs but found barriers to receipt and delivery of both physical and mental healthcare from the perspective of patients and PC practitioners. Firstly, an important and common concern for PCPs and patients is the overall lack of effective communication between PC providers and MH professionals. This can be observed through difficulties identifying a point of contact for PCPs to obtain advice & feedback from secondary MH services and disagreements regarding the overall responsibility of patients’ care. This problem was compounded by PCPs’ lack of knowledge and experience in managing SMIs which can lead to stigmatising attitudes [107, 108], another problem which was frequently discussed by both providers and service users. Secondly, a common barrier, according to patients, is providers’ inability to see beyond mental illness to take physical symptoms and concerns seriously. Diagnostic overshadowing is closely related to the evident MH stigma [109, 110] and lack of adequate knowledge around SMI and has been reported in a variety of clinical settings [111], especially during acute presentations [112]. Clinicians’ negative views of SMI patients can lead to discriminative behaviours and a lack of respect and trust towards service users, resulting in delayed presentation, missed appointments and poor adherence to treatment [113, 114]. Service users often adopt self-stigmatising views, which perpetuates their social isolation and negatively impacts their recovery [115, 116]. Therefore, addressing this MH-related stigma among PCPs is paramount for enhancing access to healthcare.

Beyond issues of access and stigma, it is important to consider the distinctive contribution of general practice as a discipline. General practitioners are uniquely positioned to manage multimorbidity, integrate physical and mental health, and deliver whole-person care. This tradition of ‘expert generalism’ has been described as a defining feature of general practice, enabling GPs to respond to the complex and interacting needs of people with SMI in ways that extend beyond disease-specific management [117, 118]. In this context, GPs are not only the first point of contact but also the clinicians best placed to provide continuous, person-centred and co-ordinated care for patients with SMI who frequently experience multimorbidity. Framing the discussion in this way underscores the importance of supporting primary care, including investment in long-term relationships and adequate resourcing, to realise the full value of general practice for this population.

It is also important to recognise that the included studies were conducted across diverse healthcare systems and cultural contexts, which may have influenced both the experiences reported and the nature of the barriers and facilitators identified. Variations in primary care organisation, funding models, and the degree of integration between mental and physical healthcare likely shaped how access was conceptualised and experienced by participants. Similarly, cultural norms surrounding mental illness, stigma, and help-seeking behaviour may have affected how service users and professionals perceived and described barriers to care. These contextual differences introduce complexity but also enhance the richness of the synthesis by highlighting the multifaceted nature of access to primary care for individuals with SMI across global settings. Recognising these contextual influences is therefore essential when interpreting the findings and considering their applicability to specific health systems.

Finally, it is important to acknowledge that most of the included primary studies did not report reflexivity practices, such as consideration of how researchers’ positionalities, professional backgrounds, or assumptions may have influenced the collection and interpretation of data [119]. This lack of reflexivity may have shaped the way participants’ voices were represented and, consequently, our synthesis. To mitigate this limitation, our review process incorporated independent coding by multiple reviewers [120], iterative team discussions to reconcile interpretations [121], and triangulation across data sources [122]. By reflecting on these potential biases and the steps taken to address them, we aimed to enhance the credibility and transparency of the review’s findings.

## Strengths and limitations of the review

To our knowledge, this review is the first conducted that collates and synthesises evidence of the perceived factors that positively or negatively influence SMI patients’ access to primary healthcare regarding their physical health. We have comprehensively searched available data sources and synthesised empirical evidence adding to the breadth and depth of knowledge regarding barriers to accessing care within PC for people with SMI. Using a mixed studies design, underpinned by a primarily pragmatic philosophical perspective, is an important strength of this review. The integration and synthesis of findings from qualitative, quantitative and mixed-method studies also facilitated a deeper understanding of the topic [123], which could not have been achieved if the topic had been explored using a single research method.

Despite our initial concerns that limited primary research would be available on the subject, we generated a desirable research portfolio, mainly thanks to our focused and systematic database search strategy, highlighting the value of the review. The strategy was developed with the contribution of a librarian specialising in healthcare research and was adapted and tested numerous times and underwent peer review [124] before it was finally used for the review searches. We employed robust screening methods including duplicate independent screening at both stages consistent with guidance [125], and reported processes in detail ensuring transparency and rigour, while reflexivity has been consistently applied throughout the review. The quality appraisal of the included papers with the use of reliable tools was also incorporated, in agreement with the consensus that quality assessment in mixed-method research enhances the credibility of the findings and the trustworthiness of the synthesis.

Several limitations should be acknowledged. Although the integration of studies employing diverse methodologies has been framed as a strength, it presented notable challenges. Synthesising research rooted in distinct philosophical and epistemological frameworks remains a relatively novel and inherently complex approach [126]. To uphold methodological rigour, consensus meetings were held for coding and data interpretation. While the search strategy was comprehensive, additional approaches—such as hand-searching relevant journals [127] or consulting field experts [128]—could have been employed to capture further pertinent studies, potentially expanding the scope of the review. Nonetheless, the focus on exhaustive database searches allowed for the prioritisation of peer-reviewed studies and those of established quality.

In addition, MeSH terms were not employed in the initial search strategy; while this decision was made to prioritise sensitivity and capture a broad range of potentially relevant studies, it may have affected the precision of the search. The inclusion of only English-language publications may have introduced language bias and limited the representation of evidence from non-English-speaking regions. The exclusion of grey literature, while intended to prioritise peer-reviewed and methodologically robust studies, may have led to the omission of relevant insights from unpublished or non-peer-reviewed sources. Furthermore, the quality and contextual diversity of included studies varied, which may influence the transferability of findings across different healthcare systems and cultural settings.

Another important limitation is the absence of direct Patient and Public Involvement (PPI) or stakeholder engagement in this review. This was primarily due to the scope and methodology of a systematic review and resource constraints. However, the study aligns with publicly identified research priorities, including NHS England Research Demand Signalling for Mental Health Nursing [129] and the James Lind Alliance Schizophrenia Priority Setting Partnership [130], which ensures relevance to the concerns of individuals with lived experience. Including direct involvement of service users and carers could have further strengthened the interpretation of findings by bringing lived-experience perspectives and highlighting priorities that may not emerge solely from the literature. Recognising this, we acknowledge the importance of integrating stakeholder perspectives and plan to incorporate structured mechanisms, such as advisory panels and stakeholder consultations, to ensure that lived experience informs interpretation and dissemination of findings in future work.

## Conclusions

### Clinical implications and future research

Our review highlights the complex interplay of factors influencing access to primary healthcare for individuals with SMI. While PCPs may encounter challenges in managing these patients, the issue extends beyond individual skills and knowledge to structural barriers, resource limitations, and the organisation of care delivery. High workload demands, time constraints, and limited integration between mental and physical healthcare services contribute to difficulties in providing equitable and comprehensive care. Ensuring that PCPs receive targeted training on mental health, including focused sessions on SMIs and approaches to shared decision-making, could help foster more effective, patient-centred interactions [20]. However, this must be accompanied by system-level changes that enable clinicians to work in conditions that facilitate high-quality, holistic care.

Furthermore, given the high prevalence of trauma among individuals with SMI, the integration of trauma-informed care within primary care represents a critical yet underexplored avenue. Trauma-informed approaches –emphasising psychological safety, trustworthiness, collaboration and sensitivity to the impact of past adversity – could improve patient engagement, reduce retraumatisation, and support recovery-oriented care. Although trauma was not explicitly addressed in the included studies, its absence highlights a significant gap in both clinical practice and research. Future work should investigate how trauma-informed principles can be systematically embedded into primary care models for people with SMI, including through staff training, adapted communication strategies, and collaborative care planning.

To improve healthcare experiences for individuals with SMI, it is essential to minimise marginalisation and healthcare disparities by ensuring that patients receive the same level of access, resources, and continuity of care as those with other chronic physical conditions. Standardising and effectively implementing annual physical health reviews across primary care settings is an important step towards reducing inconsistencies in care provision. Adherence to NHS England’s recent guidance on improving physical health in SMI should also be prioritised to drive systemic improvements.

In addition to training and structural reforms, accessible service-level practices could facilitate engagement. Our review identified potential strategies such as extended appointment durations, home visits, dedicated in-house mental health liaison roles, and flexible scheduling to accommodate the varied needs of patients with SMI. In secondary mental health services, care coordinators already play a vital role in supporting patients, yet their involvement in PC remains limited [131]. Expanding or adapting similar support structures within general practice could help bridge the gap between mental and physical healthcare, ultimately fostering a more integrated model of care [132].

Beyond clinical interventions, our findings suggest that social and community support networks play an important role in facilitating access to primary care. Patients with strong social connections—particularly support from family and friends—reported greater engagement with healthcare services [72]. Conversely, individuals experiencing social isolation, poverty, unemployment, or historical trauma often face significant barriers to accessing care. Future research should explore how community organisations, peer support networks, and formal advocacy services can provide tailored assistance to patients who lack strong personal networks, ensuring that equitable access to healthcare is maintained for all individuals with SMI.

Finally, improving communication and collaboration between primary and secondary care services remains a critical priority. Difficulties in information-sharing and unclear responsibility for patient management can hinder effective care provision. Adopting shared-information systems [77, 82, 92, 96], clearly defining care responsibilities, and designating accessible points of contact between PCPs and mental health professionals [89, 90, 92, 93] could enhance coordination and improve healthcare outcomes. Developing a structured shared-care approach, as suggested in included studies, may be instrumental in promoting holistic, multidisciplinary healthcare for individuals with SMI.

## Electronic supplementary material

Below is the link to the electronic supplementary material.

Supplementary Material 1: Table showing the characteristics of the studies that were included in the synthesis.

Supplementary Material 2: Table depicting the review’s thematic synthesis on the framework.

Supplementary Material 3

## Data Availability

All data generated or analysed during this study are included in this published article and the supplementary material. The corresponding author L.R. may be contacted for further information and for access to documents such as the data collection forms; data extracted from included studies; data used for all analyses.

## Abbreviations

JBI: Joanna Briggs Institute
MMSR(s): Mixed-Methods Systematic Review(s)
MH: Mental Health
SMI: Serious Mental Illness
PC: Primary Care
PCPs: Primary Care Professionals
PICo: Population, phenomenon of Interest & Context
PRISMA: Preferred Reporting Items for Systematic Reviews and Meta-Analyses
RCT(s): Randomised Controlled Trial(s)
SR(s): Systematic Review(s)

## Material availability

Not relevant.

## Code availability

Not relevant.

## Declarations

### Transparency declaration

The manuscript is an honest, accurate, and transparent account of the study being reported; no important aspects of the study have been omitted; and any discrepancies from the study as planned have been explained.
