# Supplementary material for: Barriers and facilitators of accessing primary healthcare for patients with severe mental illness: a mixed-methods systematic review using framework synthesis
Source: BMC Psychiatry. 2025 Nov 27;25:1131. doi: 10.1186/s12888-025-07565-x (PMC12659294; doi:10.1186/s12888-025-07565-x)
Supplement: Supplementary file 1 — Supplementary Material 1: Table showing the characteristics of the studies that were included in the synthesis. [file 12888_2025_7565_MOESM1_ESM.docx]

Table 1: Characteristics of studies that were included in the synthesis

| **Author (year) & country** | **Topic area and aim** | **Design** | **Study setting** | **Sample size** | **Participant characteristics** | | **Sampling methods** | **Data collection methods** | **Data analysis methods** |
| --- | --- | --- | --- | --- | --- | --- | --- | --- | --- |
| Awan (2020), UK | To explore the barriers and facilitators of implementing the MECC^[[1]](#footnote-1)^ approach for PCPs and patients who are under psychiatric services | Qualitative (semi-structured interviews) | GP surgeries in Lancashire | 10 patients & 10 PCPs | Patients with severe depression, paranoid schizophrenia, BD, schizoaffective disorder and personality disorder  10 GPs | | Convenience sampling of patients attending GP surgery and clinically active commissioning GPs | Individual, audio-recorded and transcribed semi-structured qualitative interviews conducted in local area | Thematic analysis |
| Bindman (1997), UK | To measure communication and joint working between psychiatric teams and GPs about a group of patients with SMI. To investigate patients' and GPs' views on GP involvement in care | Quantitative (face-to-face or telephone questionnaires) | GP surgeries in London | 100 patients (initial interview) - 90 patients (follow-up interview)  &  57 PCPs | 77/100 patients had a diagnosis of schizophrenia or schizo-affective disorder  &  57 GPs | | Convenience sampling of patients attending psychiatric outpatients in an urban geographical area and linked GP | Researcher-assisted survey questionnaires | Total satisfaction and total knowledge: student's t-tests, linear regression model. Dichotomous scores for GPs' perceived involvement and patients’ referred involvement: chi-squared tests and logistic regression. |
| Bosanquet (2010), UK | To explore views on the quality of PC for people with SMI from a dual perspective of patients and PCPs | Mixed methods (semi-structured interviews & retrospective longitudinal observational cohort) | GP surgeries in Yorkshire and Humber | 19 patients & 11 PCPs | 11 patients with BD and 8 patients with schizophrenia  7 GPs & 4 PNs | | Purposive sampling of patients using the practice QOF SMI register.   Purposive and snowballing sampling of practitioners based on clinical role & gender. | Individual, audio-recorded and transcribed, semi-structured qualitative interviews conducted in patients' homes or over the phone with PCPs | Thematic analysis |
| Burton (2015), UK | To identify current procedures, barriers and facilitators for service users, carers and health professionals to deliver CVD risk lowering interventions for people with SMI in PC | Qualitative (interviews in focus groups) | GP surgeries or community MH settings across North London, Northamptonshire, Nottinghamshire, Hampshire | 25 patients, 32 PCPs  (& 11 CMHT members, 7 carers)  *Only data from patients, PNs & GPs were included in the synthesis* | Patients with schizophrenia, schizotypal disorder, BD  16 PNs, 16 GPs | | Convenience sampling of PCPs through PC and MH clinical research networks and of patients through PC & MH clinicians | Audio-recorded and transcribed, focus groups interviews assisted by two researchers | Framework analysis |
| Butler (2020), UK | To explore the attitudes of PCPs and patients with SMI towards physical healthcare and its provision | Qualitative (semi-structured interviews) | Two CMHT: Oxfordshire Early Intervention in Psychosis Service and South Oxfordshire Adult MH team^[[2]](#footnote-2)^ | 14 patients  (& 15 MH professionals)  *Only data from patients were included in the synthesis* | Patients experiencing psychotic symptoms as part of their condition | | Purposive, convenience sampling of patients who had their care augmented by the POC device through their care coordinator | Individual, audio-recorded and transcribed, semi-structured qualitative interviews in-person or over the phone | Thematic analysis |
| Hardy (2012), UK | To examine patients' opinion of the physical health check that was delivered to them by a practice nurse who had received PhyHWell training^[[3]](#footnote-3)^ | Qualitative (semi-structured interviews) | GP surgeries in Northampton, that had nurses trained in the PhyHWell project | 5 patients with SMI | 2 patients with BD, 1 with schizophrenia, 1 with paranoid schizophrenia, 1 with a psychotic episode | | Purposive sampling of patients through GP practices | Notes and observations during meetings with heads of psychiatry & leaders), interviews with patients & social workers, focus groups with GPs, trans-sectoral focus groups, trans-sectoral workshops | Thematic analysis |
| Hassan (2020), UK | To explore the barriers and facilitators of implementing the PRIMROSE intervention into primary care across England, applying the Normalisation Process Theory to facilitate a deeper understanding of the factors that affected implementation | Qualitative (semi-structured interviews) | GP surgeries in England that delivered the PRIMROSE intervention | 15 patients with SMI  &  15 nurses and HCAs who delivered the PRIMROSE intervention | 6 patients with schizophrenia, 7 with BD, 2 with other psychosis  6 HCAs, 7 PNs, 2 research nurses (all delivered the intervention) | | Purposive sampling of patients who received the intervention and staff who delivered at least one session through GP practices | Individual, audio-recorded and transcribed, semi-structured qualitative interviews in GP practices | Thematic analysis |
| Lester (2012), UK | To explore patients’ perspectives of early-intervention services and PC in depth and over time, with a particular focus on the role each could play and on the transition process itself | Qualitative (semi-structured interviews) | Early intervention services in 5 sites across England | 63 patients took part in an initial interview, 21 agreed to a to a follow-up interview | Adults who had used early-intervention services from 5 diverse sites across England | | Convenience, maximum variation sampling strategy of patients through early-intervention services | Individual, audio-recorded and transcribed, semi-structured qualitative interviews conducted in local area at each time point, wherever possible | Harmaz's constructivist grounded theory approach |
| Lester (2005), UK | To explore the changing experience of providing and receiving PC from the dual perspectives of PCPs and patients with SMI respectively | Qualitative (focus groups) | GP surgeries in West Midlands | 45 patients with SMI  &  47 PCPs | 15 patients with schizophrenia, 4 with BD, 15 with recurrent depression, 3 with dual diagnosis, 4 with anxiety, 2 with obsessive compulsive disorder, 2 with substance misuse.  39 GPs, 8 PNs | | Snowballing sampling of patients and convenience sampling of PCPs | Audio-recorded and transcribed interviews from 18 focus groups assisted by three researchers (6 patient groups, 6 PCP groups, and 6 combined focus groups) | Thematic coding framework |
| Lester (2003), UK | To explore the concept of satisfaction with PC from the perspectives of people with schizophrenia | Qualitative (semi-structured interviews) | GP surgeries in England | 45 patients with schizophrenia | | | Random, purposive sampling of patients through the North Birmingham Community MH Localities, based on their Verona Service Satisfaction Scale score | Individual, audio-recorded and transcribed, semi-structured qualitative interviews conducted in local area | Thematic coding framework |
| Mitchell (2022), UK | To explore the perspectives of people living with SMI and Obstructive Airway Disease on their experience of respiratory healthcare in the PC setting | Qualitative (semi-structured interviews & discussion groups during a stakeholder event)  *Because <75% participants in the stakeholder discussion groups are PCPs or patients with SMI, only the interview findings were included in the synthesis* | GP surgeries in South Yorkshire | 16 patients took part in interviews.  (4 patients with SMI and 17 health/social care/third sector practitioners took part in the stakeholder event.) | Patients with schizophrenia, BD and other psychosis with comorbid asthma or COPD | | Purposive sampling of patients using standardised Systematized Nomenclature of Medicine Clinical Terms (SNOMED) coding and the UK Quality and Outcome Framework (QOF) criteria | Individual, audio-recorded and transcribed, semi-structured qualitative interviews conducted in local area | Inductive interpretive phenomenological analytical approach |
| Pitman (2011), UK | To assess current practice and attitudes related to screening and reduction in risk factors for cardiovascular disease, preferences for service provision, and perceived barriers to service uptake | Quantitative (survey) | 100 GP surgeries in Avon, North Central London, North West London | 143 PCPs,  227 SMI patients  (& 166 CMHT professionals)  *The views of CMHT professionals were not included in the synthesis* | 106 GPs, 36 PNs.  88 patients with schizophrenia, 39 with BD, 32 with other psychotic illness, 45 with depression/ anxiety, 11 with missing data. | | Random, purposive sampling of participants from areas chosen to balance urban and rural settings | Separate versions of questionnaires for patients, PCPs and CMHT staff using forced-choice response formats and Likert scales | Descriptive analysis |
| Shaw (2024), UK | To determine the acceptability of, and experiences with Primrose-A, and explore factors that influenced the implementation (including COVID-19), feasibility, and continuation of Primrose-A | Qualitative (semi-structured interviews) | London-based GP surgeries | 8 patients with SMI,  4 PCPs | 1 GP, 3 nurses,  1 peer-coach  (No further info regarding patients’ SMI diagnosis) | | Purposive sampling of patients that had previously participated in Primrose-A.   Purposive sampling of staff involved in delivering PrimroseA. | Individual, audio-recorded semi-structured interviews | Inductive reflective approach, informed by the process of thematic analysis |
| Vettini (2024), UK | To understand the challenges experienced by GPs in prescribing cardioprotective medication to patients with SMI | Qualitative (interviews) | GPs in Scotland | 15 GPs from 11 practices across 2 health board areas in Scotland | 10 GPs were from health board A and 5 from health board B;  7 practices were from health board A and 4 from health board B. | | Convenience sampling of GPs through the NRS Primary Care Network newsletter in Scotland, an email newsletter to eligible health board GPs, and contact with the ‘Deep End’ group^[[4]](#footnote-4)^ | Individual, semi-structured interviews | Braun and Clarke’s  six-phase thematic analysis |
| Wright (2006), UK | To explore the views of patients with SMI and professionals on screening for CHD risk factors and interventions for primary prevention of CHD | Qualitative (interviews) | GP surgeries in Central North London | 31 patients with SMI.  10 PCPs (& 25 CMHT professionals).  *Only the views of patients and PCPs were included in the synthesis* | 15 patients with schizophrenia, 12 with BD,  4 with schizoaffective disorder.  8 GPs & 2 PNs. | | Random, purposive sampling of participants from CMH services and GP practices in Central London | Individual, semi-structured interviews | Thematic analysis using a framework approach |
| DeCoux (2005), USA | To examine the process of health care decision making in individuals with SMI that reside in the community | Qualitative (interviews, field notes) | Residential crisis programmes that provide short-term MH care to SMI patients | 10 patients with SMI | Patients with schizophrenia, BD, major depression & chronic medical disorders (asthma, HepC, T2DM, arthritis, chronic pain due to spinal injury, CHF, hypothyroidism) | | Purposive sampling of patients from 2 residential crisis programmes in San Fransisco | Individual, audio-recorded and transcribed, qualitative interviews followed by a medical record review | Thematic analysis |
| Mangurian (2013), USA | To examine PCPs’ beliefs about the roles that PC providers and psychiatrists should play in metabolic monitoring and treatment of metabolic abnormalities among people with SMI | Quantitative (survey) | Public community health clinics in San Fransisco | 160 PCPs | 99 GPs, 57 PNs,  4 physician assistants | | Convenience sampling | Anonymous survey | Multivariate logistic regression analysis & logistic regressions |
| Smith (2017), USA | To compare differences in stigmatising attitudes across all five provider groups (PCPs, psychiatrists, psychologists, and PC and MH nurses) in a sample of VA providers | Quantitative (survey) | 5 VA hospitals in the SE and south-central areas of USA | 351 healthcare providers in total,  out of which 146 PCPs  *Only data from PCPs were included in the synthesis* | 91 PNs and 55 GPs | | Stratified random sampling of PCPs | Anonymous survey | Descriptive statistics. Three separate models were fit to examine the effects of the vignette type and provider group on the three dependent variables. All models were adjusted for four covariates: age, gender, race and years of clinical practice |
| Welch (2015), USA | To determine whether physician's attitudes toward patients with comorbid mental illness affect management of a chronic disease | Mixed methods (structured, semi-structured interviews, written chart notes) | PCPs in Massachusetts | 256 PCPs | Internists or family practitioners | | Convenience sampling | Mixed-method approach that combined structured interviews, qualitative semi-structured interviews, and written chart notes | Chi-squared tests. Standardized Cronbach's alpha. ANOVA & linear regression models. Thematic analysis (interviews) |
| Zhao (2023), USA | To explore the barriers and facilitators of primary care access among an Assertive Community Treatment team | Qualitative (interviews) | Patients and clinicians from a community MH center in Connecticut | 14 patients with SMI  (and 7 MH clinicians)  *Only data from patients were included in the synthesis* | Unclear | | Convenience sampling | Semi-structured interviews | Grounded theory approach |
| Carr (2004), Australia | To examine the attitudes and experiences of Australian GPs in the treatment of schizophrenia | Quantitative (questionnaires answered by patients and GPs) | GP surgeries in the Hunter region of New South Wales | 192 GPs  &  129 patients with schizophrenia or schizoaffective disorder | | | Purposive sampling of patients from practices the Hunter region and a volunteer schizophrenia research register. Sampling of GPs based on patients' permission to be contacted. | Two questionnaires administered to GPs. One part of one questionnaire was also completed by patients. | Group comparisons were undertaken using x2 analysis for the categorical variables (with Fisher's exact tests as required) and ANOVA for the continuous dependent variables with Scheffe follow-up comparisons. |
| Waterreus (2018), Australia | To describe from the patients’ perspective, their use of GP services over a 12-month period and the experiences, attitudes and challenges GPs face in providing this healthcare | Quantitative methods (Structured interviews with patients using a survey & questionnaires with GPs) | GP surgeries in Australia | 1825 patients interviewed  1473 GPs surveyed | Diagnosis not clear; 98 patients suffering with hallucinations, 72 with depression, 34 with mania and 33 with suicidal ideations. | | Purposive sampling of patients and GPs | Structured interviews with patients using a survey. Two questionnaires were completed by GPs | Descriptive statistics. Chi-square tests of association and analysis of variance. |
| McCabe (2008), Australia | To investigate issues related to access and barriers to health care, as well as satisfaction with health care services among individuals with schizophrenia and major depressive disorder. Views of health care providers were also obtained | Qualitative (semi-structured interviews) | Psychiatric rehabilitation centres across Melbourne and regional Victoria | 20 patients  (& 16 MH professionals)  *Only data from patients were included in the synthesis* | 10 patients with schizophrenia,  10 with major depressive disorder | | Convenience sampling of patients | Individual, semi-structured interviews with the use of a schedule that comprised 14 open-end questions | Thematic analysis |
| Spooner (2024), Australia | To identify the factors that help PWLE access preventive care from their GP to prevent long-term physical conditions | Qualitative (individual interviews and focus group) | GPs in Sydney | 20 patients  (10 in interviews and 10 in focus groups) and 5 carers | Patients with schizophrenia, BD, severe depression or anxiety disorder or other serious mental health disorder. | | Convenience sampling of patients from a publicly funded Mental Health Service and a non-government community-managed organisation | Individual face-to-faces interviews & focus groups with patients.  Family/ carer interviews conducted via telephone. | Thematic qualitative analysis using Levesque's framework |
| Jønsson (2023), Denmark | To investigate GPs' experience of the feasibility of introducing extended consultations for patients with SMI, assess the clinical content of extended consultations and investigate the feasibility of identification, eligibility screening, and recruitment of patients with SMI | Mixed methods (case report forms & observations, interviews, informal conversations, focus group) | GPs from the northern part of Jutland (5 general practices employed 7 GPs) | 38 patients with SMI & 7 GPs | 16 patients with psychotic disorder, 18 with BD,  4 with severe depression  7 GPs | | Purposive sampling of patients using the diagnostic system International Classification of Primary Care v2, followed by screening by GPs | Case reports (quantitative).  Observations of consultations,  individual semi-structured interviews,  a focus group with GPs and informal conversations with patients and GP staff (qualitative). | Interpretative phenomenological analysis |
| Jønsson (2024), Denmark | To delve into the first-hand experiences of GPs in delivering somatic care to SMI patients, concentrating on the challenges they encounter and the strategies they employ to navigate these difficulties | Qualitative (semi-structured interviews & observations) | 5 general practices in Zealand and Central Denmark | 15 GPs interviewed,  3 (out of 15) observed | Solo practice; one GP and practice staff  Company practice; more than one GP sharing facilities and staff | | Convenience sampling of GPs through an open call via the list-serve of the Research Unit for GP at the University of Copenhagen and via snowball recruiting | Semi-structured interviews and ethnographic observations | Interpretative phenomenological analysis |
| Davidsen (2020), Denmark | To explore different professionals' and patients’ experiences of trans-sectoral collaboration for patients with SMI and concurrent physical disease within the Danish health and social care system | Qualitative (notes from meetings, observations, interviews, focus groups and workshops) | GP surgeries from two Danish regions | Focus groups: 11 GPs.  Trans-sectoral focus groups: 20 healthcare professionals in total, out of which 5 GPs.  Trans-sectoral workshops: 27 healthcare professionals, out of which 7 GPs.  Interview with 1 patient in user panel. Interviews with 3 patients in social psychiatry.  *Only patient data were included as GPs <75% of the mixed professional groups* | No information given on patients’ diagnosis.  *GPs’ views not included in synthesis* | | Not described. | Qualitative: Meetings with heads of psychiatry & leaders (notes & observations), interviews with patients, interviews with social workers, focus groups with GPs, trans-sectoral focus groups, trans-sectoral workshops. | Interpretative Phenomenological Analysis (described in detail) |
| Oud (2009), Netherlands | To explore what part of the healthcare should be provided by GPs for patients with SMI in the acute and the chronic phase, whether GPs consider themselves sufficiently equipped to provide this care, how GPs manage their care for these patients in practice and their collaboration with MH services | Quantitative (survey) | GP surgeries in the Netherlands | 186 GPs across the Netherlands | | | Convenience sampling of GPs from the database of Netherlands Institute for Health Service Research | Anonymous questionnaire consisting of 20 questions | The answers on the ordinal five-level Likert scale were translated into a three-point scale, measuring either a positive or a negative response to a statement. Factor analysis & Multivariate analysis |
| Van Hasselt (2013), Netherlands | To discover what changes in the organisation of the physical health care of patients with SMI need to be made based on patients' and family carers' views | Qualitative (interviews) | PC and MH centres in the Netherlands | 10 patients with SMI (and 13 family members)  *Only data from patients were included in the synthesis* | Patients with schizophrenia, schizoaffective disorder, BD | | Convenience sampling of patients | Audio-recorded and transcribed interviews.   7 individual interviews with patients.  2 group interviews: one with 3 patients and the other with 13 family carers. | Thematic analysis |
| Collins (2021), Ireland | To report Irish service providers’ perspectives on the care of the physical health of people with SMI in an effort to inform future service developments aimed at improving the physical health of people with SMI | Qualitative (semi-structured interviews) | GP surgeries from four geolocations in Ireland | 20 PCPs  (& 14 CMHT members)  *Only data from patients were included in the synthesis* | 20 GPs | | Purposively sampling based on participants' experiences in the involvement in care of patients with SMI. | Semi-structured interviews using a topic guide | Thematic analysis |
| Bjork Bramberg (2018), Sweden | To explore the experiences and views of patients, relatives and clinicians regarding the individual and organisational factors which facilitate or hinder access to somatic healthcare for patients with SMI | Qualitative (semi-structured interviews) | GP surgeries in western Sweden | 14 patients  &  7 PCPs | 8 patients with BD, 6 patients with psychosis/ schizophrenia.  3 GPs & 4 PNs. | | Convenience sampling to achieve a diverse sample based on diagnosis, age, gender, geographical location. | Individual, audio-recorded and transcribed, semi-structured interviews | Thematic analysis. |
| Martens (2023), Belgium | To explore the perspectives of both care professionals and patients on physical healthcare in Flemish community mental services | Qualitative (semi-structured focus group & individual interviews) | GPs in Belgium | 14 patients,  7 GPs (& 5 psychiatrists and 4 CHMT professionals)  *Only the views of patients and PCPs were included in the synthesis* | 3 patients with schizophrenia, 2 with BD,  5 with schizoaffective disorder, 1 with psychotic depression, 2 with EUPD, 1 with substance abuse.  7 GPs | | Random, convenience sampling of patients through an invitation letter.  Convenience sampling of GPs via invitation email and snowballing. | Individual, semi-structured interviews with patients  Online semi-structured mixed focus group with GPs (and psychiatrists).  Individual interviews for physicians unable to attend the focus group. | Thematic analysis |
| Jego (2019), France | To better understand GPs' views attitudes and needs in the care of patients with mental disorders | Qualitative (semi-structured interviews) | GP surgeries in Marseille, France | 22 GPs working various areas in Marseille  (50% of them estimated that >30% of their patients suffered from mental disorders) | | | Purposeful variation sampling of GPs, to obtain a diversified sample on gender, experience, office organisation, interest in MH and exposure to mental disorders | Initial ethnographic phase when investigator observed and recorded GPs' consultations. Individual, recorded and transcribed, semi-structured interviews using an interview guide. Field notes were made during and after the interviews. | Thematic analysis |
| Kapungwe (2011), Zambia | To document negative and/or positive types of attitudes of PCPs towards people suffering from mental illness and possible predictors of such attitudes | Quantitative (questionnaires answered by health care providers) | GP surgeries in Lusaka and Mumbwa | 111 participants in total:  95 PCPs  (& 16 MH professionals) | | 32 general clinical officers, 26 Zambia registered nurses, 33 Zambia enrolled nurses, 4 environmental health technologists | Convenience sampling | Anonymous survey | Simple cross-tabulations were used to calculate proportions and their distributions in different groups |
| Sahile (2019), Ethiopia | To assess the attitude of PC nurses and its associated factors towards people with SMI in Addis Ababa | Quantitative (questionnaires) | Primary health practices in Addis Ababam Ethiopia | 610 PNs | | | Simple random sampling technique | Structured self-administered questionnaire | Descriptive statistics. Bivariate and multivariate binary logistic regression analysis. |
| Lavie-Ajayi (2018), Israel | To examine the GP-patient interface by employing a dual perspective guided by the Capability Approach framework, in order to understand the barriers that hinder the provision of physical healthcare for people with SMI | GP surgeries from a range of geographical regions and socio-economic profiles in Israel | GP surgeries from a range of geographical regions and socio-economic profiles in Israel | 10 PCPs  &  15 patients with SMI | 10 GPs  &  9 patients with schizophrenia and 6 with BD | | Purposive sampling of GPs and patients from a range of geographical regions and socio-economic profiles | Semi-structured interviews, with the use of two different interview guides, one for each party | Systematic content and thematic analysis |
| O’Brien (2021), New Zealand | (To measure compliance with a metabolic monitoring protocol within secondary MH services of the DHB.) To assess PNs’ perceptions of their role in physical health monitoring of MH consumers | Quantitative (survey) & two audits.  *Only the survey results were included in the synthesis, as the audits were not directly answering the review’s research question* | Practices in New Zealand's southern district health board | 24 PNs from the southern district health board in New Zealand | | | Convenience sampling | Anonymous survey | Descriptive statistics. The survey responses were categorised into variables of knowledge, attitudes, practice and PC systems. |
| Vaccari (2020), Chile | To determine how stigmatisation occurs towards people diagnosed with SMI in PC, based on the experiences of those affected and PCPs | Qualitative (semi-structured interviews & discussion groups) | PC services in Concepcion & Talcahuano | Two patient groups: 12 patients each (24 patients in total).  Two PCP groups: 6 and 7 people (13 PCPs in total).  Semi-structured interviews: 5 patients in total | Patients with SMI & PCPs  (No additional information provided.) | | Convenience sampling | Discussion groups & semi-structured interviews | Thematic analysis using researcher's triangulation. |

**Key:** BD: Bipolar Disorder PN(s): Practice Nurse(s)

CHD: Congenital Heart Disease PC: Primary Care
 CHF: Congestive Heart Failure PCP: Primary Care Professionals

CMHT: Community Mental Health Team PIL: Participant Information Leaflet

EUPD: Emotionally Unstable Personality Disorder POC: Point of Care

HCA: Healthcare Assistant T2DM: Type 2 Diabetes

HPC: Hepatitis C

MHP: Mental Health Professionals

1. Making Every Contact Count (MECC) is an approach to behaviour change that capitalises upon these routine interactions between patients and health professionals to encourage positive change to physical health and mental wellbeing. [↑](#footnote-ref-1)
2. Forms part of a larger mixed-methods evaluation of a POC device in CMHT. Not set in primary healthcare but examining SMI pts and community MH professionals’ ideas regarding patients' physical healthcare and its provision. Only patients’ opinions will be included. [↑](#footnote-ref-2)
3. The Northampton Physical Health and Wellbeing (PhyHWell) project is a training package for PNs that aims to influence the nurses' role and as a result improve patient outcomes. [↑](#footnote-ref-3)
4. ‘Deep End’ refers to the GP practices serving the 100 most deprived populations in Scotland. [↑](#footnote-ref-4)
