# Supplementary material for: Barriers and facilitators of accessing primary healthcare for patients with severe mental illness: a mixed-methods systematic review using framework synthesis
Source: BMC Psychiatry. 2025 Nov 27;25:1131. doi: 10.1186/s12888-025-07565-x (PMC12659294; doi:10.1186/s12888-025-07565-x)
Supplement: Supplementary file 2 — Supplementary Material 2: Table depicting the review’s thematic synthesis on the framework. [file 12888_2025_7565_MOESM2_ESM.docx]

Table 2: Thematic synthesis on framework

| **Access-to-healthcare conceptual framework** | | | | | | | | | |
| --- | --- | --- | --- | --- | --- | --- | --- | --- | --- |
| Approachability | Acceptability | Availability & Accommodation | Affordability | Appropriateness | Ability to perceive | Ability to seek | Ability to reach | Ability to pay | Ability to engage |
| **Rapport between patient & clinician**  -Clinician’s communication skills  -Trust, comfort & respect  **Continuity of care**  -Empathy and encouragement  -Established connections  **Stigma by clinicians &**  **self-stigmatisation**  -Fear & discomfort by clinicians  -Lack of respect & empathy | **Holistic care**  **Active patient participation in their care** | **Time constraints**  **-**Length of consultations  **Overworking of staff, lack of support and resources**  -Funding cuts  **Availability/ length of appointments & other practical issues**  -Proactive invitations & structured reviews  -Atmosphere of waiting rooms, excessive administrative procedures, etc.  -Accommodation regarding location/ timing  -Accessible contact information | **Incentivised/ free services** | **Lack of communication & cooperation between MH & PC professionals**  -Disagreements between clinicians regarding responsibilities  -Patients’ confusion  -Poor continuity of knowledge between clinicians  **PCPs’ competence regarding managing SMI patients**  -Lack of knowledge & sense of inadequacy  -Lack of confidence  -Appropriate training  **Diagnostic overshadowing**  **Hopelessness regarding diagnosis**  -SMI as life-sentence | **Patient-related factors**  -Cognitive skills  -Lack of health awareness  -Communication difficulties  -Being accompanied during appointments  **Clinician-related factors**  -Jargon  -Patient education  -Patient-tailor-made interventions | **Poor appointment attendance**  **Lack of trust to clinicians**  **Lack of confidence because of SMI**  **Advocacy** | **Housing instability**  **Transportation**  **Social isolation** | **Insurance**  **Costs when attending** | **SMI as a barrier**  -MH instability  -Communication difficulties  -Stress & lack of motivation  -Negative symptoms  **Poverty & unemployment**  **Social support vs social isolation**  - Advocacy  **Housing instability**  **Achievable goals** |
