# Supplementary material for: Barriers and facilitators of accessing primary healthcare for patients with severe mental illness: a mixed-methods systematic review using framework synthesis
Source: BMC Psychiatry. 2025 Nov 27;25:1131. doi: 10.1186/s12888-025-07565-x (PMC12659294; doi:10.1186/s12888-025-07565-x)
Supplement: Supplementary file 3 — Supplementary Material 3 [file 12888_2025_7565_MOESM3_ESM.docx]

## Supplementary material

Table s1: PRISMA 2020. Statement and checklist.

| **Section and Topic** | **Item #** | **Checklist item** | **Location where item is reported** |
| --- | --- | --- | --- |
| **TITLE** | | |  |
| Title | 1 | Identify the report as a systematic review. | Title |
| **ABSTRACT** | | |  |
| Abstract | 2 | See the PRISMA 2020 for Abstracts checklist. | Abstract |
| **INTRODUCTION** | | |  |
| Rationale | 3 | Describe the rationale for the review in the context of existing knowledge. | Introduction |
| Objectives | 4 | Provide an explicit statement of the objective(s) or question(s) the review addresses. | Introduction |
| **METHODS** | | |  |
| Eligibility criteria | 5 | Specify the inclusion and exclusion criteria for the review and how studies were grouped for the syntheses. | Methods |
| Information sources | 6 | Specify all databases, registers, websites, organisations, reference lists and other sources searched or consulted to identify studies. Specify the date when each source was last searched or consulted. | Methods |
| Search strategy | 7 | Present the full search strategies for all databases, registers and websites, including any filters and limits used. | Methods |
| Selection process | 8 | Specify the methods used to decide whether a study met the inclusion criteria of the review, including how many reviewers screened each record and each report retrieved, whether they worked independently, and if applicable, details of automation tools used in the process. | Methods |
| Data collection process | 9 | Specify the methods used to collect data from reports, including how many reviewers collected data from each report, whether they worked independently, any processes for obtaining or confirming data from study investigators, and if applicable, details of automation tools used in the process. | Methods |
| Data items | 10a | List and define all outcomes for which data were sought. Specify whether all results that were compatible with each outcome domain in each study were sought (e.g. for all measures, time points, analyses), and if not, the methods used to decide which results to collect. | Methods |
|  | 10b | List and define all other variables for which data were sought (e.g. participant and intervention characteristics, funding sources). Describe any assumptions made about any missing or unclear information. | Methods |
| Study risk of bias assessment | 11 | Specify the methods used to assess risk of bias in the included studies, including details of the tool(s) used, how many reviewers assessed each study and whether they worked independently, and if applicable, details of automation tools used in the process. | Methods |
| Effect measures | 12 | Specify for each outcome the effect measure(s) (e.g. risk ratio, mean difference) used in the synthesis or presentation of results. | N/A |
| Synthesis methods | 13a | Describe the processes used to decide which studies were eligible for each synthesis (e.g. tabulating the study intervention characteristics and comparing against the planned groups for each synthesis (item #5)). | Methods |
|  | 13b | Describe any methods required to prepare the data for presentation or synthesis, such as handling of missing summary statistics, or data conversions. | Methods |
|  | 13c | Describe any methods used to tabulate or visually display results of individual studies and syntheses. | Methods |
|  | 13d | Describe any methods used to synthesize results and provide a rationale for the choice(s). If meta-analysis was performed, describe the model(s), method(s) to identify the presence and extent of statistical heterogeneity, and software package(s) used. | Methods |
|  | 13e | Describe any methods used to explore possible causes of heterogeneity among study results (e.g. subgroup analysis, meta-regression). | Methods |
|  | 13f | Describe any sensitivity analyses conducted to assess robustness of the synthesized results. | Methods |
| Reporting bias assessment | 14 | Describe any methods used to assess risk of bias due to missing results in a synthesis (arising from reporting biases). | Methods |
| Certainty assessment | 15 | Describe any methods used to assess certainty (or confidence) in the body of evidence for an outcome. | N/A |
| **RESULTS** | | |  |
| Study selection | 16a | Describe the results of the search and selection process, from the number of records identified in the search to the number of studies included in the review, ideally using a flow diagram. | Results |
|  | 16b | Cite studies that might appear to meet the inclusion criteria, but which were excluded, and explain why they were excluded. | Supplementary materials |
| Study characteristics | 17 | Cite each included study and present its characteristics. | Results/ Table 1 |
| Risk of bias in studies | 18 | Present assessments of risk of bias for each included study. | Results/  Tables s6 & s7 |
| Results of individual studies | 19 | For all outcomes, present, for each study: (a) summary statistics for each group (where appropriate) and (b) an effect estimate and its precision (e.g. confidence/credible interval), ideally using structured tables or plots. | N/A |
| Results of syntheses | 20a | For each synthesis, briefly summarise the characteristics and risk of bias among contributing studies. | Results |
|  | 20b | Present results of all statistical syntheses conducted. If meta-analysis was done, present for each the summary estimate and its precision (e.g. confidence/credible interval) and measures of statistical heterogeneity. If comparing groups, describe the direction of the effect. | N/A |
|  | 20c | Present results of all investigations of possible causes of heterogeneity among study results. | N/A |
|  | 20d | Present results of all sensitivity analyses conducted to assess the robustness of the synthesized results. | N/A |
| Reporting biases | 21 | Present assessments of risk of bias due to missing results (arising from reporting biases) for each synthesis assessed. | N/A |
| Certainty of evidence | 22 | Present assessments of certainty (or confidence) in the body of evidence for each outcome assessed. | N/A |
| **DISCUSSION** | | |  |
| Discussion | 23a | Provide a general interpretation of the results in the context of other evidence. | Discussion |
|  | 23b | Discuss any limitations of the evidence included in the review. | Discussion |
|  | 23c | Discuss any limitations of the review processes used. | Discussion |
|  | 23d | Discuss implications of the results for practice, policy, and future research. | Discussion |
| **OTHER INFORMATION** | | |  |
| Registration and protocol | 24a | Provide registration information for the review, including register name and registration number, or state that the review was not registered. | Methods |
|  | 24b | Indicate where the review protocol can be accessed, or state that a protocol was not prepared. | Methods |
|  | 24c | Describe and explain any amendments to information provided at registration or in the protocol. | N/A |
| Support | 25 | Describe sources of financial or non-financial support for the review, and the role of the funders or sponsors in the review. | Declarations |
| Competing interests | 26 | Declare any competing interests of review authors. | Declarations |
| Availability of data, code and other materials | 27 | Report which of the following are publicly available and where they can be found: template data collection forms; data extracted from included studies; data used for all analyses; analytic code; any other materials used in the review. | Declarations |

Table s2: PRISMA 2020. Abstract checklist.

| **Section and Topic** | **Item #** | **Checklist item** | **Reported (Yes/No)** |
| --- | --- | --- | --- |
| **TITLE** | | |  |
| Title | 1 | Identify the report as a systematic review. | Yes |
| **BACKGROUND** | | |  |
| Objectives | 2 | Provide an explicit statement of the main objective(s) or question(s) the review addresses. | Yes |
| **METHODS** | | |  |
| Eligibility criteria | 3 | Specify the inclusion and exclusion criteria for the review. | Yes |
| Information sources | 4 | Specify the information sources (e.g. databases, registers) used to identify studies and the date when each was last searched. | Yes |
| Risk of bias | 5 | Specify the methods used to assess risk of bias in the included studies. | Yes |
| Synthesis of results | 6 | Specify the methods used to present and synthesise results. | Yes |
| **RESULTS** | | |  |
| Included studies | 7 | Give the total number of included studies and participants and summarise relevant characteristics of studies. | Yes |
| Synthesis of results | 8 | Present results for main outcomes, preferably indicating the number of included studies and participants for each. If meta-analysis was done, report the summary estimate and confidence/credible interval. If comparing groups, indicate the direction of the effect (i.e. which group is favoured). | Yes |
| **DISCUSSION** | | |  |
| Limitations of evidence | 9 | Provide a brief summary of the limitations of the evidence included in the review (e.g. study risk of bias, inconsistency and imprecision). | Yes |
| Interpretation | 10 | Provide a general interpretation of the results and important implications. | Yes |
| **OTHER** | | |  |
| Funding | 11 | Specify the primary source of funding for the review. | No |
| Registration | 12 | Provide the register name and registration number. | No |

Table s3: Search strategy

| Population |  | Interest |  | Context |
| --- | --- | --- | --- | --- |
| Serious Mental Illness OR  Severe Mental Illness OR  Severe Mental Disorder OR Serious Mental Disorder OR  SMI OR  Schizophrenia OR  Psychosis OR  Psychotic disorder OR  Bipolar* | AND | Access OR  Screening OR  Treatment OR  Interv* | AND | Primary Care OR  Primary Healthcare OR  Primary Health Care OR  GP OR  General Practice |
| AND Observation* OR Perspective* OR View* OR Experience* | | | | |

Table s4: Search results by database

| **Database (provider)** | **Citations identified  (1^st^ round of searches)** | **Citations identified  (2^nd^ round of searches)** | **Total no. of citations** |
| --- | --- | --- | --- |
| Medline (Ovid) | 396 | 131 | 527 |
| Embase (Ovid) | 859 | 52 | 911 |
| PsycINFO (Ovid) | 446 | 215 | 661 |
| Web of Science | 3799 | 522 | 4321 |
| CINAHL Plus (EBSCO) | 377 | 22 | 399 |
| **Total** | **5877** | **942** | **6819 (incl. duplicates)** |

Table s5: Study eligibility criteria

**Inclusion:**

- Studies that report on access to and provision of treatment for physical healthcare for people with SMI within primary care services and explore the views, experiences, attitudes and beliefs of SMI patients and healthcare professionals working in primary care centres.
- Eligible populations:
  - SMI patients (patients diagnosed with schizophrenia, psychotic disorders, bipolar disorder and depression with psychotic features).
  - Primary healthcare professionals (GPs, practice nurses, healthcare assistants and other healthcare professionals who are employed in primary care centres).
- Where populations are mixed and data cannot be disaggregated such that extracting data for the population of interest only is not possible, studies will be included if > 75% of the population of interest are included.

**Exclusion:**

- Studies in non-English language will not be included.
- Secondary sources such as summaries, brief reports, book chapters, conference abstracts, editorials/ opinion or discussion pieces/ commentaries and literature reviews will be excluded.
- Studies not using empirical data, studies that have not been peer-reviewed, grey literature, study protocols and unpublished work will also be excluded.
- Studies with a population of <14 years of age will not be included.
- Systematic and scoping reviews will not be included.
- Studies conducted in dental practices or involve dentists, medical students or pharmacists will not be included.

If the full text is not available, the authors will be conducted and a period of 2 weeks will be allowed for a reply. If access to the full text is still not gained after 2 weeks, the article will be excluded.

Table s6: Quality appraisal of qualitative research

| Author & year | Q1 | Q2 | Q3 | Q4 | Q5 | Q6 | Q7 | Q8 | Q9 | Q10 | Overall appraisal: |
| --- | --- | --- | --- | --- | --- | --- | --- | --- | --- | --- | --- |
| Awan 2020 | Unclear | Yes | Yes | Yes | Yes | Yes | Yes | Yes | Yes | Yes | Include |
| Bjork Bramberg 2018 | Yes | Yes | Yes | Yes | Yes | Yes | Unclear | Yes | Yes | Yes | Include |
| Bosanquet 2018 | Yes | Yes | Yes | Yes | Yes | Yes | Yes | Yes | Yes | Yes | Include |
| Burton 2015 | Unclear | Yes | Yes | Yes | Yes | Unclear | No | Yes | Yes | Yes | Include |
| Butler 2020 | Unclear | Yes | Yes | Yes | Yes | No | No | Yes | Yes | Yes | Include |
| Collins 2021 | Unclear | Yes | Yes | Yes | Yes | No | No | Yes | Yes | Yes | Include |
| Davidsen 2020 | Unclear | Yes | Yes | Yes | Yes | Yes | No | Yes | Yes | Yes | Include |
| DeCoux 2005 | Unclear | Yes | Yes | Yes | Yes | No | No | Yes | Unclear | Yes | Include |
| Hardy 2012 | Unclear | Yes | Yes | Yes | Yes | Unclear | No | Yes | Yes | Yes | Include |
| Hassan 2020 | Unclear | Yes | Yes | Yes | Yes | Unclear | Yes | Yes | Yes | Yes | Include |
| Jego 2019 | Unclear | Yes | Yes | Yes | Yes | Unclear | Yes | Yes | Yes | Yes | Include |
| Jønsson 2023 | Unclear | Yes | Yes | Yes | Yes | No | No | Yes | Yes | Yes | Include |
| Jønsson 2024 | Unclear | Yes | Yes | Yes | Yes | Yes | No | Yes | Yes | Yes | Include |
| Lavie-Ajayi 2018 | Unclear | Yes | Yes | Yes | Yes | No | No | Yes | Yes | Yes | Include |
| Lester 2012 | Unclear | Yes | Yes | Yes | Yes | Yes | Yes | Yes | Yes | Yes | Include |
| Lester 2005 | Unclear | Yes | Yes | Yes | Yes | Yes | Unclear | Yes | Yes | Yes | Include |
| Lester 2003 | Unclear | Yes | Yes | Yes | Yes | Yes | Unclear | Yes | Yes | Yes | Include |
| Martens 2023 | Unclear | Yes | Yes | Yes | Yes | Yes | Yes | Yes | Yes | Yes | Include |
| McCabe 2008 | Unclear | Yes | Yes | Yes | Yes | No | No | Yes | Unclear | Yes | Include |
| Mitchell 2022 | Unclear | Yes | Yes | Yes | Yes | No | No | Yes | Yes | Yes | Include |
| Shaw 2024 | Unclear | Yes | Yes | Yes | Yes | Yes | Yes | Yes | Yes | Yes | Include |
| Spooner 2024 | Unclear | Yes | Yes | Yes | Yes | Yes | No | Yes | Yes | Yes | Include |
| Vaccari 2020 | Unclear | Yes | Yes | Yes | Yes | No | No | Yes | Yes | Yes | Include |
| vanHasselt 2013 | Unclear | Yes | Yes | Yes | Yes | Yes | Yes | Yes | Yes | Yes | Include |
| Vettini 2024 | Unclear | Yes | Yes | Yes | Yes | No | No | Yes | Yes | Yes | Include |
| Welch 2015 | Unclear | Yes | Yes | Yes | Yes | Yes | Unclear | Yes | Unclear | Yes | Include |
| Wright 2006 | Unclear | Yes | Yes | Yes | Yes | No | No | Yes | Yes | Yes | Include |
| Zhao 2023 | Unclear | Yes | Yes | Yes | Yes | No | Unclear | Yes | Yes | Yes | Include |

Key for Table s6

Q1. Is there congruity between the stated philosophical perspective and the research methodology?

Q2. Is there congruity between the research methodology and the research question or objectives?

Q3. Is there congruity between the research methodology and the methods used to collect data?

Q4. Is there congruity between the research methodology and the representation and analysis of data?

Q5. Is there congruity between the research methodology and the interpretation of results?

Q6. Is there a statement locating the researcher culturally or theoretically?

Q7. Is the influence of the researcher on the research, and vice- versa, addressed?

Q8. Are participants, and their voices, adequately represented?

Q9. Is the research ethical according to current criteria or, for recent studies, and is there evidence of ethical approval by an appropriate body?

Q10. Do the conclusions drawn in the research report flow from the analysis, or interpretation, of the data?

Table s7: Quality appraisal of quantitative research

| Author & year | 1. Were the criteria for inclusion in the sample clearly defined? | 2. Were the study subjects and the setting described in detail? | 3. Was the exposure measured in a valid and reliable way? | 4. Were objective, standard criteria used for measurement of the condition? | 5. Were confounding factors identified? | 6. Were strategies to deal with confounding factors stated? | 7. Were the outcomes measured in a valid and reliable way? | 8. Was appropriate statistical analysis used? | Overall appraisal |
| --- | --- | --- | --- | --- | --- | --- | --- | --- | --- |
| Bindman 1997 | Yes | Yes | Yes | Unclear | Yes | Yes | No | Yes | Include |
| Bosanquet 2018 | Yes | Yes | Yes | Yes | Yes | Yes | Yes | Yes | Include |
| Carr 2004 | No | Yes | Yes | Yes | Unclear | Unclear | Yes | Yes | Include |
| Jønsson 2023 | Yes | Yes | Unclear | Yes | No | No | Unclear | Unclear | Include |
| Kapungwe 2011 | No | Yes | Unclear | N/A^[[1]](#footnote-1)^ | Unclear | No | Unclear | Yes | Include |
| Mangurian 2013 | Yes | No | Unclear | N/A | No | Yes | Yes | Yes | Include |
| O'Brien | Yes | Yes | Unclear | N/A | No | No | Unclear | Unclear | Include |
| Oud 2009 | Yes | No | Unclear | N/A | No | No | Unclear | Yes | Include |
| Pitman 2011 | No | Yes | Unclear | Unclear | No | No | Unclear | Unclear | Include |
| Sahile 2019 | Yes | Yes | Yes | N/A | Yes | Yes | Unclear | Yes | Include |
| Smith 2017 | No | Yes | Yes | N/A | Yes | Yes | Yes | Yes | Include |
| Waterreus 2018 | No | Unclear | Yes | Yes | No | No | Yes | Yes | Include |
| Welch 2015 | Yes | Yes | Unclear | N/A | Yes | Yes | Yes | Yes | Include |

Box s1. Approachability: quotations

"If the clinician does not have good communications skills and causes a negative experience for a patient, they’re not going to want to come to the doctors for anything" (Patient) (1)

"Rapport was considered as half the battle or probably more" (PCP) (1)

"[I] used to see a different doctor every time and they would just say, “oh, try this, oh that hasn’t worked, I’ll tell you what … try this.” It was pot luck" (Patient) (8)

"If there’s a blood draw... she gives me this paper and [I] go to the lab and get a blood draw... Very fast follow-up... I’m there maybe... ten minutes... But waiting is like five hours... and then they say, “Bye.”... I don’t even feel I’m okay. She’s doing my follow-ups like she... doesn’t even care... She just prescribes the medication... and that’s it.” (Patient) (15)

"I feel that health care professionals should be more understanding and empathetic towards people with a mental illness. Many of them do not understand how to relate to you when they know that you have schizophrenia." (Patient) (7)

"He [the GP] has never taken an interest and asked me, “how do you feel?” Maybe there is a need for one less pill. She just prescribes the medication, and I take [the prescription] to the pharmacy." (Patient) (12)

"When continuity is present, you’re going to know that they’re not just bringing it up, just for hell of it, they’re doing it for the best" (Patient) (1)

"Continuity of staff is vital, it’s absolutely vital. And especially for the weaker members of society, continuity is incredibly important for them. It’s more important than anything else, actually, and the health centre which has had the same staff for a long time, where there’s good continuity, well it obviously works much better, and when the doctor has known the patients since way back, then there’ll be fewer problems, so continuity is incredibly important." (PCPs) (11)

"I’ve got a good doctor now … I picked him, because he was the only one who helped me … he believes me." (Patient),

"SMI patients] are always keen to have their own GP, so they would rather wait." (PCP)

(8)

“One of the problems in hospital is that every time you go you see a different doctor and you have to tell your story all over again. I have such a good relationship with my GP, and it’s much better because I don’t have to start from the beginning each time. It’s really quite painful going over the story again and again and it’s much better just to have one person who knows you.” (Patient) (13)

"Making them feel comfortable by making a relationship with them to start with... And that is making a relationship with them to come back and encouraging them in their own way.” (PCP) (5)

“You've got a familiar face who knows your story and you don't have to start from the beginning again. She's seen me deteriorate and come back again. I feel very safe in her hands.” (Patient) (10)

"Regardless of the wait, I’m willing to wait for [my nurse practitioner] because she’s quick. She listens to what I have to say and she’s very good at what she does. And I can talk to her about many issues as far as my hepatitis C or my pancreas problem or this having a wisdom tooth pulled and with an infection in my sinus canal, have a CT scan done and not have any medical insurance" (Patient) (15)

Box s2. Acceptability: quotations

"For me, a care package would be designed around eating, sleep patterns and physical activity, and that would help …a whole person, for the wellbeing of all the body …rather than just cater for them highs and lows. You can mend your head but if you can’t mend everything else there’s no point in mending that, is there? But I don’t think that’s going to happen sometime soon because they haven’t got the time, have they?" (Patient) (8)

(The essence of general practice should be) “a continuity of holistic care not just your mental health.” (PCP) (1)

“From my view is that it’s (the intervention) very much based on a holistic approach of the patient. So it’s patient-centric and in looking at everything whereas a normal GP appointment is five minutes and it’s transactional…” (Patient, referring to intervention studied) (5)

“She thinks that if I see a psychiatrist then everything is …even if I come to her [i.e., the GP] then everything is a psychiatric [problem].” (Patient) (12)

“I think my other practitioners had ignored (symptoms of fibromyalgia) because of my mental health problem.” (Patient) (1)

“I think that you need to be familiar with where the person is coming from; his family, what helps him to take his medications and remain stable, what knocks him off balance and… even to be a bit like a kindergarten teacher, to tell them, to ask them, to remind them, not to expect them to do it all alone. But also on the other hand, to give them autonomy, [to] give them autonomy and allow them to make decisions for themselves, give them the dignity as a person like anyone else, not to treat just the mental illness but see the person as a whole.” (PCP) (12)

“If I am taking tablets, I want to know about them, about the side effects, things like that. I like to know what is going on. I should be part of that decision.” (Patient) (13)

“I felt like a zombie and I didn’t want any more injections and he said they would come to the house and give it to me if I didn’t turn up. So I just kept on having them even though I felt terrible.” (Patient) (13)

“It would be extremely useful if my GP took the time to provide me with information that I can use to make informed choices about my health. I would have been in much better physical health now if I had known that my diet can influence my dental health as well as my mental health.” (Patient) (7)

Box s3. Availability and accommodation: quotations

“And if you could do one thing in that appointment, you had to choose the one thing, because you knew you weren’t going to get the whole … you’re not going to be able to work through your template. You think, ‘Well, if I can get this blood pressure today, or I can do one thing today, then I’ve got somewhere." (PCP) (3)

“I'm not sure how it would fit in easily in a surgery that's already quite packed. We've got ever-growing lists, so whether it could be done in more of a mental health environment, it may be more appropriate..." (PCP, referring to the intervention studied) (5)

“Previously (author: before the introduction of the right to choose) you never talked about medical priorities in relation to what the diagnosis was and what you got money for. But today everything is steered by which patients and diagnoses are registered at the health centre. You should really… If you haven’t got enough time for everything, you should remove what you don’t get money for. We don’t actually say that, but that’s the reality.” (PCP) (11)

“I’d love to do an annual check and to have a dedicated half hour session every six months. But I don’t have the time, I’m trying to see 35 or 40 patients a day in 10- or 15-minute slots so to try with something as complex as somebody with EMI who also may have co- morbid illnesses and polypharmacy, is by far the biggest problem I have” (PCP) (14)

“GPs are all overworked we try and fight fire and you prioritise things, this I suspect you say right where is patients with mental health physical health on your priority list and I think it would be pretty low near the bottom...” (PCP) (1)

“I did feel as if I was on my own a little bit in the surgery... There was just not support as in, I'm worried about this patient, but it was maybe just reading consultation notes, that sort of thing” (PCP) (5)

“I've always got backup. I wouldn't have hesitation in asking any of the senior and qualified staff. I think we've got the backup here to do a really good job” (PCP) (5)

“The focus is on risk assessment. But why do you have to be in crisis before you get help? You need to kick up a stink” (Patient) (23)

“Where they’re perhaps not very good is that I tend to be the one who has to remind (the GP practice) about my yearly MOT, as I like to call it. Now, I don’t think that’s very good.” (Patient) (3)

"But sometimes I can’t get there because I’ve got appointments with the hospital. But hospital, she likes me to be there so I cancel that and I say ‘can you make me another one." (Patient) (6)

“One morning when I went there to get some exams done, I waited a long time but the girl didn’t call me. Another woman was called before me so I said to the girl, it’s my turn now, not that lady’s turn, and I left and went home feeling sad” (Patient - Consequences of a long waiting time) (2)

“Another person was referred to a dietician. As I said, that was in regard to his cholesterol, and he was referred to a specialist, as well. And I think that probably happened a bit more quickly than it might have done... I think we're quite lucky, out here, that you can refer people on different groups, clubs...” (PCP) (5)

"The clinician’s role was felt to be an ‘agent of change" (PCP) (1)

"I would like to know the results as soon as possible. So you don’t have to brood for two weeks if everything will be all right." (Patient) (17)

"Something, finding slots when we're so busy, that, that would be a thing as well, so sometimes you think to yourself, well, you know, this patient needs extra time, but actually we haven't got a slot... to fit her in" (PCP) (5)

“The good ones you know, they tend to have good systems too so you can get to see the GP when you need to easily.” (Patient) (20)

Box s4. Affordability: quotations

“We don’t have a smoking cessation service in general practice anymore. That’s been defunded. There is a council driven one, and I don’t know how effective – or active – it is. But we’re specifically not funded to provide any smoking cessation advice other than to tell people generally about it." (PCP) (8)

“One of our patients I was trying to refer for a local scheme, actually the funding has been cut, for weight reduction.” (PCP) (3)

Box s5. Appropriateness: quotations

“The communication, in terms of letters from clinics and things is terrible, often waiting months to get a letter about medication changes. By the time you get the letter, the medication has changed again, so that’s really difficult.” (PCP) (8)

"We don’t have a great relationship with secondary care. I mean if a patient has got a CPN … we don’t have any records particularly of who their CPN is unless they’ve got that information in a letter. And we don’t have any regular contact with anybody’s CPN" (PCP) (8)

"So I go to the bipolar clinic, you know that, and then I get sent to the emergency unit, and at the emergency unit they say no, your heart’s beating slowly because of your medication. So I go back to the bipolar clinic and “No, I don’t think so because the dose is so low”. Ok. You can never get the whole picture, it’s always just bits like this, so you just have to try to piece it together yourself." (Patient) (11)

“The information comes through the patient, usually verbally… or half the time the patient can’t remember what they were told to get done” (PCP - Referring to lack of communication with CMHT) (14)

“I don't see that I would contact my coordinator for my physical health. I think that's more the mental side of things. The GP would make more sense for the first port of call.” (Patient) (18)

"I was using medication from my psychiatrist, but my GP was totally uninformed." (Patient) (17)

“For psychotic patients it’s different. The psychiatrist takes the lead" (PCP) (22)

“Psychiatrists appear to function in secret…That they should not tell us anything and that we should not ask. As if we were not ourselves of this medical word […]. Sometimes we don’t know the patient’s disorder and we don’t have a letter from the psychiatrist. As if it was a secret.” (PCP) (22)

“We have brilliant mental health nurses who we can contact very readily if we have concerns. It’s a difficult part of medicine but it actually runs very well in comparison to other chronic illness” (PCP) (14)

“There are, let’s say, diabetes reports, blood pressure reports, I can… see who are my diabetes patients and who did not do the test, this is very important to the management (…) But I don’t have a report of schizophrenic patients, so…but… there are indirect ways, if I have a patient who is both schizophrenic and diabetic and he didn’t do the test so… [I can say] “Ah! I really haven’t seen him for long time, let’s get his file and see what has happened.” (PCP) (12)

"Continuity of staff is vital, it’s absolutely vital. And especially for the weaker members of society, continuity is incredibly important for them. It’s more important than anything else, actually, and the health centre which has had the same staff for a long time, where there’s good continuity, well it obviously works much better, and when the doctor has known the patients since way back, then there’ll be fewer problems, so continuity is incredibly important." (PCPs) (11)

“If the key worker brings them, they might be happy to come along, so that’s another way of trying to get access to them.”,

“I like the fact that it would be probably long term, and the fact that you hopefully would see the same person.” (PCPs)

(3)

“In the past we thought that it will be good if we can have a psychiatrist in the clinic (…) I need to know how to treat him [a patient with SMI], and how to cope with him and so on. How to solve the mental together with the physical difficulties, how to cooperate with him better, this is the aim.” (PCP) (12)

“Maybe you don’t have good enough specialist knowledge about these groups (author: patients) and the problems they can have, what we do in principle is treat them like any other patient. Then you usually get, can get problems with communication, or compliance, and then we usually get help from a psychiatric consultant.” (PCP) (11)

“I know that I cannot look after people with severe and enduring mental health problems. I do not have the skills or the knowledge. I couldn't do it well.” (PCP) (10)

“There was a mental health update I went to as part of my PDP, but there are no updates on that [SMI]. We have lots of things for dementia wise, but not with serious mental illness.” (PCP) (8)

“Some people find these patients threatening or scary or difficult. Depending on the practice and the expertise you've got within it, there are bound to be training needs there just in terms of making people feel comfortable with patients who present with more challenging behaviours or difficulties. If you're frightened of a patient, and lots of professionals who don't work in mental health care, and they can be scary, and therefore, it's much easier not to have the difficult conversation about the fact that, yes, okay, they're on medications, but they really could do a bit more to help themselves by losing weight or stopping smoking or not drinking so much.” (PCP) (3)

“There has never really been a, as you say a workshop to evaluate and differentiate, as she says, what mental health really is…” (PCP) (2)

“We had to use HCAs who maybe didn't have quite as much training...” (Patient) (9)

“As I said (name), you want people that are more qualified on the topic of mental health, mental health. Once we attended a group… uh… for mental health… they need more capable people… people who understand… what mental health is all about” (Patient) (2)

“I think it's always good to have somebody whose kind of responsibility it is to make sure that things happen... I mean, if there was funding. That person wouldn't necessarily do it all but they would kind of have an overall responsibility for making sure that it, you know, happened and coming round to the practices and saying, "What are you doing?" and checking our records, or whatever, to find out” (PCP) (16)

Box s6. Ability to perceive: quotations

“I can see extra needs, these particular patients can be more difficult to assess and, in some cases, can be dissimulating, that they don’t express the morbidity they may have in the same way other people do. There’s a risk that you don’t take their complaints as seriously, I think, there’s a risk of making light of them in some way. I worked in psychiatric care for a while before I became a doctor. That’s why I’m extra cautious in a way.” (PCP) (11)

“It depends on the degree or the severity of the illness on how much insight they have really. Some of them might manage quite well. There’s such a spectrum. A lot of them just behave like any other patient, they attend whenever they are worried about their blood pressure or they have a pain in their back or leg or tummy” (PCP) (14)

“I think using the book for something like that, you do need a lot of time to go through it with them... I think maybe the book made it feel too formulised... I don't find that book a very easy layout so I think that was almost a stumbling block. Maybe I didn't understand the book particularly and the pts didn't particularly find it helpful” (PCP) (5)

“It’s never been brought up to me that there was a link between mental health and heart disease; until I was telling the psychiatric nurse that I was coming to this. She said, ‘Oh, yes, you’re more likely to develop heart trouble,’ etc. And I thought, ‘Well, nobody had …’ I mean, surely the doctor should pinpoint that, and perhaps do an examination once a year to see how your heart is going on and whether you’ve developed diabetes or anything in the meantime.” (Patient) (3)

“I’m not personally that worried, but because I was here and it was, you know, it’s part of the service I was quite happy to do [the physical health check]. It’s good to find out the results but I wouldn’t like to go out my way to go find out about it.” (Patient) (18)

“Sometimes, yeah. And then sometimes you think well you might as well be speaking in a foreign language because I don't understand anything. If you come out with…if people use big terms, big medical terms, I don't understand, no. Speak so I can understand it.” (Patient) (6)

“I don't know what to ask for, so would like to be offered more information about services available to me” (Patient) (4)

" Because my Dad's got Diabetes… I thought there was a risk that I could have it.. But the good thing about having that physical health check is to make sure if I've got it or not". (Patient) (18)

"The clinician’s role was felt to be an ‘agent of change" (PCP) (1)

Box s7. Ability to seek: quotations

“The system at this doctor means you see (a) different doctor or nurse each time. Not good when you have mental illness and cannot trust people.” (Patient) (4)

“It’s like I've got (name of carer), I've got a couple of other friends, you know, that if I'm feeling, you know, like, at night time, they’d rally round. She’s a trained social worker, but she doesn’t work for social services, … and she’s connected with the doctors, she works with the National Health, so that was the difference. She was compassionate.” (Patient) (6)

Box s8. Ability to reach: quotations

“When I get sick it's really hard to get to my doctor. I can't travel by public transport, because usually I get very confused about what's around me and am not able to ask for help.” (Patient) (7)

“I get stressed out sort of thing. I spend most of my life in here now, where you wouldn't get me in the house at one bit.” (Patient) (6)

Box s9. Ability to engage: quotations

“Quite a lot of them [SMI patients] just don’t turn up, despite repeated letters and things, and telephone calls. They just sort of decline to, more so than normal patients … they’re just a challenging cohort of patients really, they often aren’t engaging particularly well and it is more difficult to manage them.” (PCP) (8)

“Their failure to attend is so high that they do slip through the system. Because you’re sending out a letter, aren’t you, and a lot of them just don’t attend.” (PCP) (3)

"We can deal with somebody with diabetes and all of that, and we can tell them this, that and the other, but somebody with mental health, when they’ve got that problem, they may not have that understanding. They may not engage for a long period of time. It’s really very difficult… I prefer people who can engage with me.", (PCP)

"... it was the patients that didn't come. You just get frustrated; you put all of this time and effort into the first appointment and then you never saw them again." (PCP)

(5)

“She is at home a lot. It is hard to get her out of the house. Last week, I took her for a stomach ultrasound, and it was very difficult. I had to set an alarm for 9 am, to wake her up and wash her and dress her, and to go out in the heavy traffic. It was raining, I had to find parking and find the place in the building and wait in line and [get to] the ultrasound room, and it was difficult, it was very difficult.” (Patients’ husband) (19)

“If one were to bring up lifestyle intervention in this stage it could give the impression that I am not listening.” (PCP),

“Mental health patients as a cohort are more vulnerable and a lot of them, there is a reliance on the GP to guide them.” (PCP)

(1)

“It may be for good reasons or it may be from prejudice, that you feel, 'I can't remember the last time I managed to get a patient with really significant mental health problems to stop smoking.' Part of me thinks, 'This person's life is miserable. They have a very difficult life and smoking is the one thing they can do.” (PCP) (3)

“At the end, what’s going to happen? Am I going to be able to keep my flat or is this going to get worse so am I’m unable to look after myself? I don't think it will…I think it’ll come to the point where I have to go in some kind of supported housing just so I've got…like if anything happened to me at night nobody would know.” (Patient) (6)

“Now I'm trying to start looking after myself in terms of eating healthily and not too much, and I've started swimming, and I do walk a bit. But it's trying to build it up slowly. I'm not really finding that the weight is coming off particularly easily now.” (Patient) (3)

“Definitely, they (carer) come to appointments with me, talk to the doctor, talk to the nurse, be involved in a very real way.” (Patient) (3)

“Once I went with my father. That time the attention was good, but when I go by myself it’s different.” (Patient),

“I have tried to turn this situation around. I try to be more sociable so that people like me, I try to get along better with them” (Patient)

(2)

“It would be extremely useful if my GP took the time to provide me with information that I can use to make informed choices about my health. I would have been in much better physical health now if I had known that my diet can influence my dental health as well as my mental health.” (Patient) (7)

“Identifying one key behaviour change, it’s really important to give them achievable goals and be realistic.” (PCP) (3)

Box s10. Stigma-related barriers: quotations

“I think my other practitioners had ignored (symptoms of fibromyalgia) because of my mental health problem.” (Patient) (1)

“When I came in, they couldn’t even do tests at the emergency clinic. So it was a real emergency. But what I mean, what I want to get to, is that the reason I just hung around at home so long is that I’m scared they’re going to say “You’ve got these problems because of your bipolar diagnosis and the side effects of your medication.” You’re scared of ever going to the doctor’s, because that’s absolutely not what you want thrown at you.” (Patient) (11)

“I would just like to be taken seriously when you go. I always go and they say things like, 'Well, it could be connected to your mental health or you're feeling anxious … ' and then you go away and you're back next week because you've still got the same symptoms. At the moment, I feel well enough to actually challenge that, but if you're feeling low, you don't have the wherewithal to actually say, 'It's not my anxiety. I know my body. I know it's not right.” (Patient) (3)

"I was a bit disappointed. I went in one day with a bad chest and it was for a mental health check and it was a psychiatrist and she was very nice, but I was annoyed because they didn’t tell me that. I went because I thought it was for my chest because I wanted some help. And she listened to my chest and she said ‘oh it’s a bit rough but you'll be better in two months’ time, it’ll be completely alright’ and I thought that was strange saying that." (Patient) (21)

"My contacts with my doctor have been useful since I changed doctors. But before, my doctor did not read his file properly and even though I was not progressing physically, he did not review his treatment. He continued to put me on these medications until my health deteriorated to the point that I requested another opinion." (Patient) (7)

"She thinks that if I see a psychiatrist then everything is… even if I come to her [i.e., the GP] then everything is a psychiatric [problem]." (Patient)

"I think that there is [a tendency] to put less effort in preventive medicine and also in the treatment of the psychiatric patient, to deal less with complaints and symptoms. It is easier to think that these complaints are not genuine – not genuinely physical – even though they are. That’s my general feeling." (PCP)

(12)

“…even the ones that have their mental health symptoms very much under control, they tend to focus on the mental health symptoms and not so much the physical health symptoms.” (PCP) (14)

“About these aggressions, for example, we’ve had service users, I remember last year when a secretary didn’t give, didn’t give a patient an appointment, and he left and slashed the car tires, so there is a feeling of vulnerability” (PCP) (2)

“I’d just signed on with this new GP um and my papers hadn’t come through so I told him I’d just come out of (hospital) ... and he looked really worried and asked me if I’d been on a section and said he wasn’t sure he could be my doctor and maybe I should find another surgery.” (Patient) (13)

“I think a lot of this fear that you're saying is coming around the idea that they're suddenly going to have a morning full of unstable patients appearing at their doorway.” (PCP) (3)

“I don’t know, but they always tell me “You’re here again!”, they say. The paramedics, another paramedic said to me “Why are you here again? You were just here not too long ago…” (Patient),

“If I go by myself, they don’t pay attention to me because they say that a person like me can’t show up alone to receive medical attention. They say that a responsible adult has to be with me.” (Patient),

“So, since they see us differently, because we have an illness, it’s like they brush us off to the side and for me everybody is the same, they have the same rights, mentally ill or normal, it’s like… you make an appointment, you need to be punctual, to have more patience, and calmness to treat that person because he got up early to stand in line, and what’s more, he gets rejected, they ignore him and others go before him; in the end, all CESFAMs are a drag.” (Patient)

(2)

"They really class it (mental illness) as a taboo subject and, you know, that you must be of a lower class and whatever.” (Patient) (6)

“I was a bit shocked when he first told me I wasn’t going to get better. Later on he told me I’d be sick for a long long time. I wanted to tell him that I was feeling a bit better.” (Patient) (13)

“He was great, a really good GP. He didn’t put blocks in my way and say I couldn’t do things. He said, ‘well if you can get the qualifications and you’re well enough then you should go ahead and do it.” (Patient) (13)

References

1. Awan H, Ditta MA, McKeown M, Whittaker K. 'A positive thing by mentioning it': a qualitative study of experiences of brief physical health interventions for individuals diagnosed with severe mental illness in primary care. World Fam Med. 2020;18(10):84-90.

2. Vaccari P, Ramirez-Vielma R, Saldivia S, Cova F, Vielma-Aguilera A, Victoriano V, et al. Stigma towards people with a diagnosis of severe mental disorder in primary healthcare centers: Perspectives of service users and health teams in Chile. International Journal of Mental Health Systems Vol 14 2020, ArtID 6. 2020;14.

3. Burton A, Osborn D, Atkins L, Michie S, Gray B, Stevenson F, et al. Lowering Cardiovascular Disease Risk for People with Severe Mental Illnesses in Primary Care: A Focus Group Study. Plos One. 2015;10(8):16.

4. Hardy S, Deane K, Gray R. The Northampton Physical Health and Wellbeing Project: The views of patients with severe mental illness about their physical health check. Mental Health in Family Medicine. 2012;9(4):233-40.

5. Hassan S, Heinkel S, Burton A, Blackburn R, McCloud T, Ross J, et al. A qualitative study exploring the barriers and facilitators of implementing a cardiovascular disease risk reducing intervention for people with severe mental illness into primary care contexts across England: the 'PRIMROSE' trial. Bmc Health Services Research. 2020;20(1):15.

6. Mitchell C, Zuraw N, Delaney B, Twohig H, Dolan N, Walton E, et al. Primary care for people with severe mental illness and comorbid obstructive airways disease: a qualitative study of patient perspectives with integrated stakeholder feedback. BMJ Open. 2022;12(3):e057143.

7. McCabe MP, Leas L. A qualitative study of primary health care access, barriers and satisfaction among people with mental illness. Psychology, Health & Medicine. 2008;13(3):303-12.

8. Bosanquet K, Gilbody S, Watt I, Shiers D, Coventry P, Owen C. Closing the mortality gap: Meeting the physical health needs of people with serious mental illness (SMI) in primary care. Early Intervention in Psychiatry. 2018;12(Supplement 1):230.

9. Hassan S, Ross J, Marston L, Burton A, Osborn D, Walters K. Exploring how health behaviours are supported and changed in people with severe mental illness: A qualitative study of a cardiovascular risk reducing intervention in Primary Care in England. Br J Health Psychol. 2020;25(3):428-51.

10. Lester H, Tritter JQ, Sorohan H. Patients' and health professionals' views on primary care for people with serious mental illness: focus group study. Bmj. 2005;330(7500):1122.

11. Bjork Bramberg E, Torgerson J, Norman Kjellstrom A, Welin P, Rusner M. Access to primary and specialized somatic health care for persons with severe mental illness: a qualitative study of perceived barriers and facilitators in Swedish health care. BMC Family Practice. 2018;19(1):12.

12. Lavie-Ajayi M, Moran GS, Levav I, Porat R, Reches T, Goldfracht M, et al. Using the capabilities approach to understand inequality in primary health-care services for people with severe mental illness. Israel Journal of Health Policy Research. 2018;7(1):49.

13. Lester H, Tritter JQ, England E. Satisfaction with primary care: The perspectives of people with schizophrenia. Family Practice. 2003;20(5):1-11.

14. Collins C, Finegan P, O'Shea M, Larkin J, Pericin I, Osborne B. Promoting physical health among people with enduring mental illness: a qualitative study of healthcare providers' perspectives. BMJ Open. 2021;11(4):6.

15. DeCoux M. Acute versus primary care: The health care decision making process for individuals with severe mental illness. Issues in Mental Health Nursing. 2005;26(9):935-51.

16. Wright CA, Osborn DP, Nazareth I, King MB. Prevention of coronary heart disease in people with severe mental illnesses: A qualitative study of patient and professionals' preferences for care. BMC Psychiatry Vol 6 2006, ArtID 16. 2006;6.

17. van Hasselt FM, Oud MJT, Loonen AJM. Improvement of care for the physical health of patients with severe mental illness: a qualitative study assessing the view of patients and families. Bmc Health Services Research. 2013;13:7.

18. Butler J, de Cassan S, Turner P, Lennox B, Hayward G, Glogowska M. Attitudes to physical healthcare in severe mental illness; a patient and mental health clinician qualitative interview study. BMC Family Practice. 2020;21(1):243.

19. Kapungwe A, Cooper S, Mayeya J, Mwanza J, Mwape L, Sikwese A, et al. Attitudes of primary health care providers towards people with mental illness: evidence from two districts in Zambia. African Journal of Psychiatry. 2011;14(4):290-7.

20. Lester H, Allan T, Wilson S, Jowett S, Roberts L. A cluster randomised controlled trial of patient-held medical records for people with schizophrenia receiving shared care. British Journal of General Practice. 2003;53(488):197-203.

21. Mitchell C, Zuraw N, Delaney B, Twohig H, Dolan N, Walton E, et al. Primary care for people with severe mental illness and comorbid obstructive airways disease: a qualitative study of patient perspectives with integrated stakeholder feedback. BMJ Open. 2022;12(3):e057143.

22. Jego M, Debaty E, Ouirini L, Carrier H, Beetlestone E. Caring for patients with mental disorders in primary care: a qualitative study on French GPs' views, atittudes and needs. Family Practice. 2019;36(1):72-6.

23. Lester H, Tritter JQ. 'Listen to my madness': understanding the experiences of people with serious mental illness. Sociology of Health & Illness. 2005;27(5):649-69.

1. No patients were included as participants. [↑](#footnote-ref-1)
